# Impacts of Chalcogen Bonding on the Stability and Reactivity of 5‐Iminothianthrene Platform: Toward Electrophilic Nitrogen Sources

**DOI:** 10.1002/chem.202501045

**Published:** 2025-05-13

**Authors:** Ryutaro Tawara, Shohei Hamada, Takumi Furuta, Yusuke Kobayashi

**Affiliations:** ^1^ Department of Pharmaceutical Chemistry Kyoto Pharmaceutical University 1 Misasagishichono‐cho, Yamashina‐ku Kyoto 607‐8412 Japan; ^2^ Division of Pharmaceutical Sciences Graduate School of Medical Sciences Kanazawa University Kakuma‐machi Kanazawa 920‐1192 Japan

**Keywords:** cyanamide, metathesis, nitrene, noncovalent interactions, photo‐irradiation

## Abstract

The thianthrene scaffold has recently attracted significant attention in various coupling reactions. Although 5‐iminothianthrene and its derivatives are expected to function as electrophilic aminating reagents, their properties and reactivity have not been fully investigated. In this work, it was found that 5‐iminothianthrene reacted with various electrophiles, such as acid chlorides, protons, and isothiocyanates to give the corresponding adducts. In addition, intramolecular chalcogen bonding (ChB) was identified in those adducts, and this interaction was found to contribute to their stability and reactivity. Finally, the potential of *N*‐acyliminothianthrenes as *N*‐acylnitrene equivalents was demonstrated.

## Graphical abstract

5‐Iminothianthrene reacted with various electrophiles, such as acid chlorides, protons, and iso(thio)cyanates, to afford the corresponding adducts. Intramolecular chalcogen bonding (ChB) was identified through natural bond orbital (NBO) analysis based on their X‐ray crystal structures. The potential of these adducts as electrophilic aminating reagents was demonstrated.

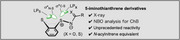

## 1 Introduction

**Scheme 1:**
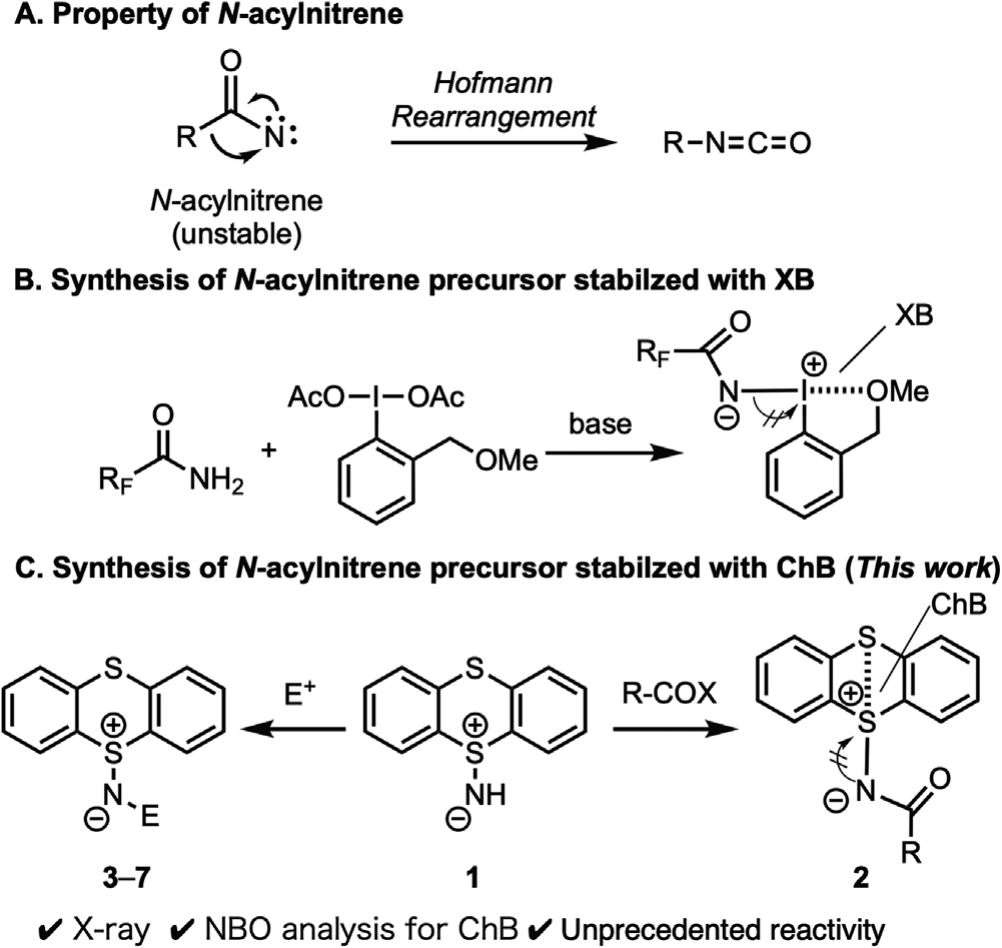
Background and summary of this work.

Chalcogen bonding (ChB) is a net attractive noncovalent interactions (NCIs) between an electrophilic region associated with a chalcogen atom in a molecular entity and a nucleophilic region in another, or the same, molecular entity.^[^ ^1^ ^]^ ChB has recently attracted significant attention in various research fields, such as catalysis and molecular recognition.^[^ ^2^, ^3^, ^4^ ^]^ However, a different application of ChB, including conformational control of molecules, is an emerging research area, and only a limited number of examples have been reported.^[^ ^5^, ^6^, ^7^, ^8^ ^]^ We envisioned that ChB could be utilized for the stabilization of potentially unstable molecules, as we have recently reported that halogen bonding (XB),^[^ ^9^, ^10^, ^11^, ^12^ ^]^ another type of NCIs, is effective in stabilizing and identifying carbene^[^ ^13^, ^14^ ^]^ and *N*‐acylnitrene precursors (Scheme 1).^[^ ^15^, ^16^, ^17^, ^18^ ^]^ Although nitrenes are among the most useful species for introducing nitrogen atoms into various molecules^[^ ^19^ ^]^ and facilitating skeletal editing,^[^ ^20^ ^]^ *N*‐acylnitrenes have been less explored due to their instability toward Hofmann rearrangement and/or hydrolysis (Scheme 1A),^[^ ^21^ ^]^ in contrast to *N*‐sulfonylnitrenes.^[^ ^22^ ^]^ Only *N*‐trifluoroacetyl and *N*‐perfluoroalkanoyl derivatives have been synthesized through the condensation of relatively acidic amides with iodoarene diacetate under basic conditions (Scheme 1B).^[^ ^15^, ^17^ ^]^ We envisioned that various *N*‐acylnitrene equivalents could be readily synthesized from 5‐iminothianthrene (**1**),^[^ ^23^ ^]^ which is readily accessible from thianthrene,^[^ ^24^, ^25^, ^26^ ^]^ and that intramolecular ChB would prevent Hofmann rearrangement. Only a limited number of studies have explored on the reactivity of **1** with electrophiles,^[^ ^27^ ^]^ and no crystallographic structures of *N*‐acyliminothianthrene **2** have been reported to date. We reportin this study the structural data of **2** with different acyl groups, as well as the inter‐ and intramolecular interactions based on structural analysis and density functional theory (DFT) calculations (Scheme 1C). In addition, we investigated the reactivity of **1** toward various electrophiles to obtain the adducts **3**–**7**, and unprecedented reactivity of the adducts was observed attributed to intramolecular ChB interactions. We believe that the properties and reactivity of **1**–**7** are valuable for the design and development of a new class of electrophilic aminating reagents.

## 2 Results and Discussion

**Figure 1:**
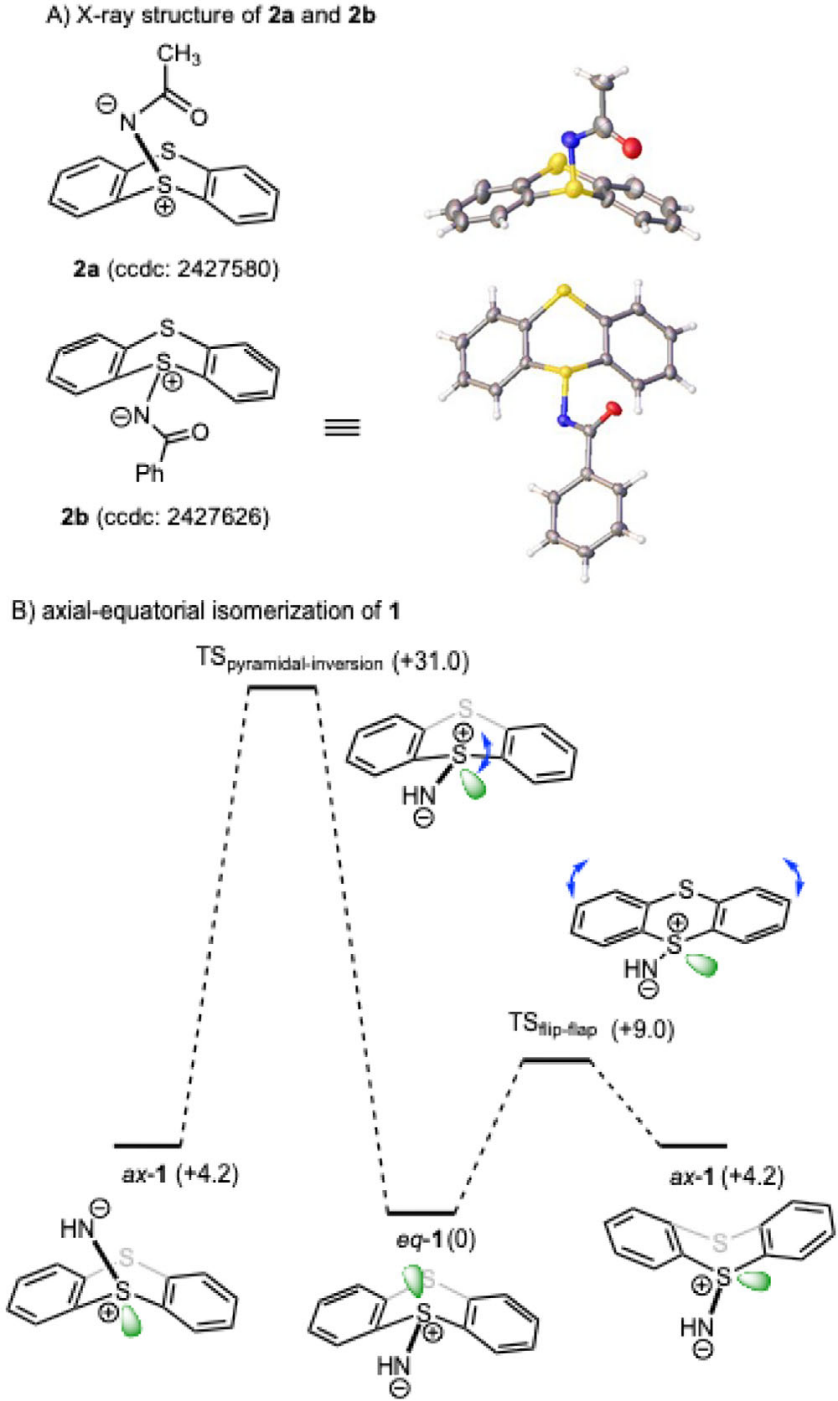
(A) X‐ray structure of **2a**,**b**, and (B) axial‐equatorial isomerization of **1**.

We first reacted **1** with several acylating reagents under standard acylation conditions (Table 1). Overall, the acylated products **2a**–**e** were obtained in good to high yields (71%–94%), and fortunately recrystallization of **2a**–**e** afforded suitable crystals for X‐ray structural analysis (Figure 1A and Tables S1,S2).

**Table 1:** Acylation of 5‐iminothianthrene **1**.

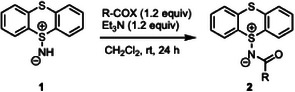				
Entry	R	X	Product	Yield (%)^[^ ^a^ ^]^
1	CH_3_	Cl	**2a**	88
2	Ph	Cl	**2b**	71
3	4‐MeOC_6_H_4_	Cl	**2c**	94
4	CF_3_	OCOCF_3_	**2d**	84
5^[^ ^b^ ^]^	OBu^t^	OCO_2_Bu^t^	**2e**	75

^[a]^ Isolated yield.

^[b]^ Without Et_3_N.

**Figure 2:**
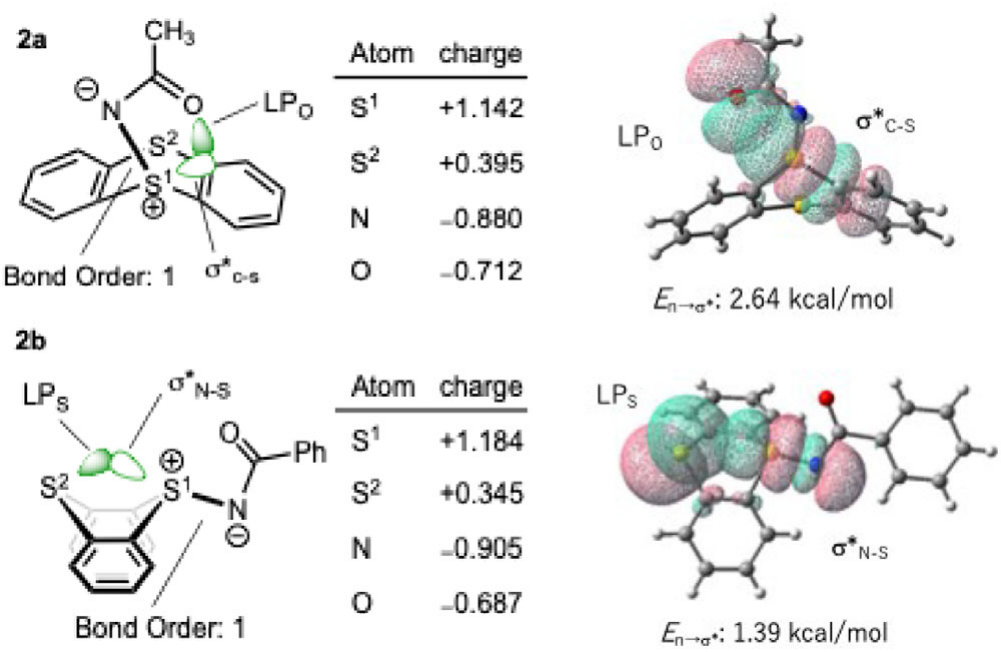
NBO analysis of **2a** and **2b**.

X‐ray structure analysis revealed that the *N*‐acetyl substituent of **2a** was positioned axially, whereas the other *N*‐acyl variants (**2b**–**e**) adopted an equatorial position. It is known that the axial and equatorial forms of thianthrene derivatives are interconvertible around the S–S axis of the dithiin framework via the so‐called “flip‐flap” isomerization, rather than through axial‐equatorial interconversion via thermal pyramidal inversion at approximately 100 °C.^[^ ^28^, ^29^ ^]^ In fact, we performed DFT calculations^[^ ^30^ ^]^ to investigate the axial/equatorial interconversion processes of **1** and **2** (Figure 1B and Figure S1), and found that the activation energy for the transition state (TS) of flip‐flap isomerization of **1** is 9.0 kcal/mol, whereas that for the TS of pyramidal inversion exceeds 30 kcal/mol. Since the equatorial form is thermodynamically more stable than the axial form, presumably due to steric factors, the axial form of **2a** appears to be the kinetic product and exhibits superior crystallinity compared to the equatorial form. To gain insight into the properties, natural bond orbital (NBO) analysis of **2a** was performed (Figure 2). The bond order of the N─S bond was found to be 1.00, and the NBO charges of the sulfur and nitrogen atoms were +1.14 and −0.880, respectively. The negative charge of the nitrogen atom was delocalized onto the carbonyl oxygen atom, while the positive charge of the sulfur atom (S^1^) was delocalized over the other sulfur atom (S^2^). Noting that intramolecular ChB was identified between the lone pair of carbonyl oxygen atom and the σ* orbital of the S─C bond with interaction energies of 2.64 kcal/mol. In addition, intermolecular ChB (LP_S_ → σ*_S─N_) was also observed in the axial form of **2a** (Table S3), which likely contributes to its good crystallinity. Furthermore, NBO analysis of the equatorial form of *N*‐acyliminothianthrene, such as **2b**, revealed the presence of intramolecular ChB (LP_S_ → σ*_S─N_), which might inhibit the formation of *N*‐acylnitrene and the subsequent Hofmann rearrangement, thereby facilitating the easy isolation of *N*‐acylnitrene precursor **2**. In addition, an intramolecular ChB between the lone pair of carbonyl oxygen atom and the σ* orbital of the S─C bond was also observed (Table S4).

**Scheme 2:**
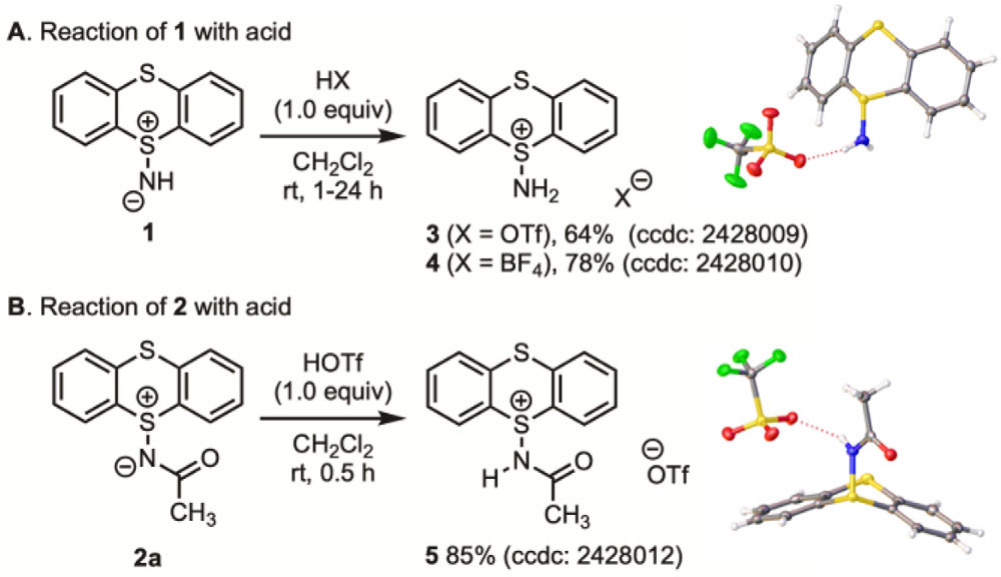
Reaction of **1** and **2** with acid.

We next investigated the protonation of **1** and **2** to enhance their potential as electrophilic nitrogen sources (Scheme 2). Protonation of **1** with trifluoromethanesulfonic acid (TfOH) proceeded smoothly, affording the corresponding triflate salt **3** in 64% yield, while the reaction with HBF_4_ afforded the corresponding salt **4** in 78% yield (Scheme 2A). In addition, *N*‐acyliminothianthrene **2** also reacted with TfOH, yielding the corresponding triflate salt **5** in 85% yield (Scheme 2B). The structures of **3**–**5** were unambiguously determined by spectral data and X‐ray crystallographic analysis (Table S5). However, compounds **3**–**5** were found to undergo gradual hydrolysis at ambient temperature, whereas compound **2** could be stored for several months without noticeable degradation.

We further investigated the reactivity of **1** with different electrophiles in the presence of Et_3_N, and found that the reaction with phenyl isocyanate and phenyl isothiocyanate proceeded smoothly, affording the corresponding adducts **6a** and **7a** in 70% and 80% yields, respectively (Table 2, entries 1 and 2). As the solubility of **7a** in common organic solvents, such as CHCl_3_, CH_2_Cl_2_, and EtOAc, was significantly higher than that of **6a**, the scope of this reaction was explored using various isothiocyanates (entries 3–7). Both electron‐donating and electron‐withdrawing groups on the aromatic ring of phenyl isothiocyanate did not affect the reactivity with **1**, and thiourea‐type products **7b** and **7c** were obtained in high yields (entries 3 and 4). Substitutions at the ortho‐ and meta‐positions, as well as functional groups such as Br, CF_3_, and the pyridine ring, were well tolerated under the optimized conditions, affording the adducts **7d**–**f** in 64%–83% yields (entries 5–7).

**Table 2:** Reaction of 5‐iminothianthrene **1** with iso(thio)cyanate.

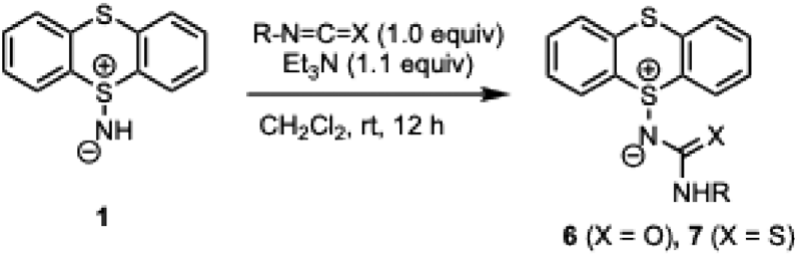				
Entry	R	X	Product	Yield(%)^[^ ^a^ ^]^
1	Ph	O	**6a**	70
2	Ph	S	**7a**	80
3	4‐MeOC_6_H_4_	S	**7b**	75
4	4‐NO_2_C_6_H_4_	S	**7c**	88
5	2‐BrC_6_H_4_	S	**7d**	72
6	3,5‐(CF_3_)_2_C_6_H_3_	S	**7e**	64
7	2‐pyridyl	s	**7f**	83

^[a]^ Isolated yield.

**Figure 3:**
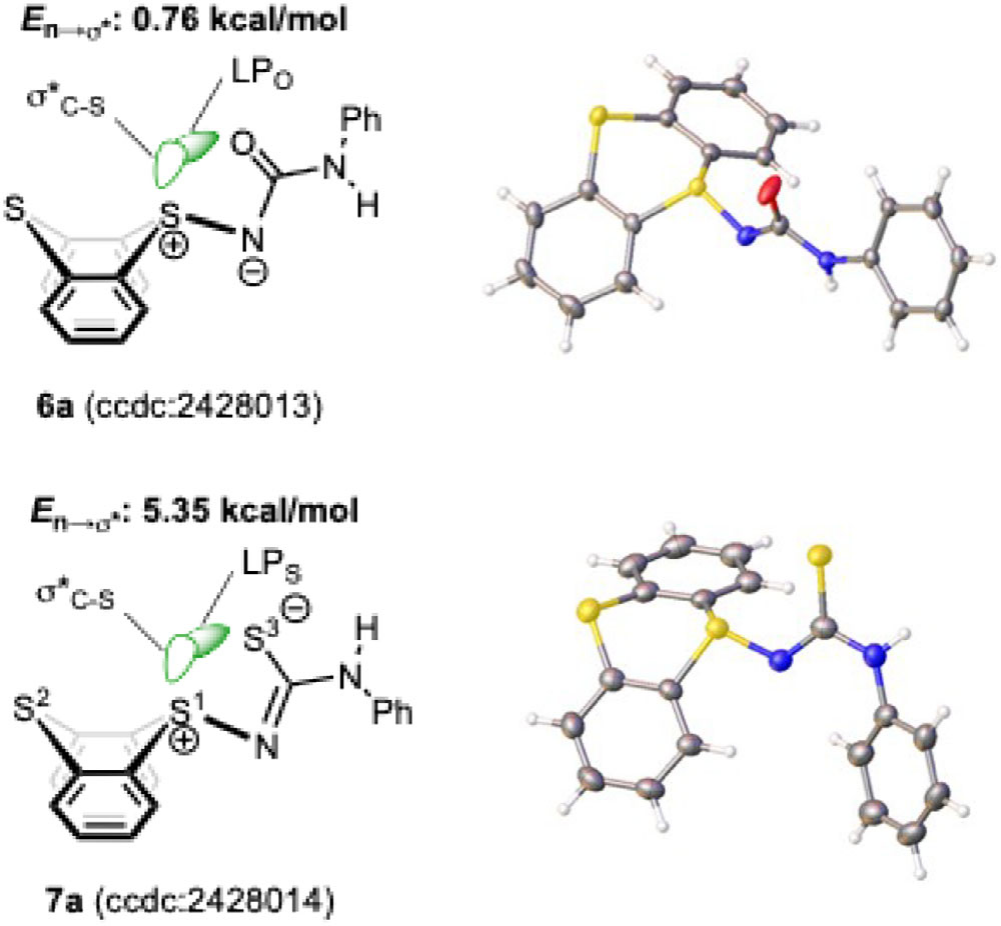
NBO analysis of **6a** and **7a** based on their X‐ray crystal structures.

It is worth mentioning that intramolecular ChB was observed in both **6a** and **7a**, as evidenced by X‐ray crystallographic analyses (Table S6) and subsequent NBO analysis (Figure 3 and Tables S7,S8). The interaction energy of **7a** between the lone pair of the sulfur atom in the thiocarbonyl group and the σ* orbital of S─C bond in the thianthrene scaffold was estimated to be 5.35 kcal/mol (LP_S3_ → σ*_S1─C_). This interaction energy was significantly higher than that observed in **6a** (LP_O_ → σ*_S─C_, 0.76 kcal/mol). The higher interaction energy in **7a** can be attributed to the greater polarizability of the sulfur atom^[^ ^31^ ^]^ in the thiocarbamoyl moiety compared to the oxygen atom in the carbamoyl group of **6a**.

Notably, the gradual degradation of **7** was observed during the collection of the ^13^C NMR spectrum, and the mass spectra exhibited [M‐248]^+^ signals, suggesting that **7** degrades with the loss of thianthrene (216) and one sulfur atom (32). We assumed that this degradation was caused by intramolecular ChB, followed by a metathesis‐type reaction,^[^ ^18^ ^]^ leading to the formation of cyanamide **8**, which can serve as an outstanding building block as well as a ligand for transition metals.^[^ ^32^ ^]^ In fact, when **7a**–**e** were heated in dichloroethane at 80 °C, complete conversion into the corresponding cyanamides **8a**–**e** was achieved in good to high yields within 2 hours (Table 3).

**Table 3:** Thermal transformation of **7** into cyanamide **8**.

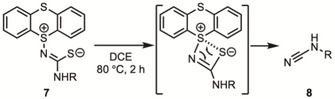			
Entry	R	Product	Yield (%)^[^ ^a^ ^]^
1	Ph	**8a**	79
2	4‐MeOC_6_H_4_	**8b**	65
3	4‐NO_2_C_6_H_4_	**8c**	93
4	2‐BrC_6_H_4_	**8d**	89
5	3,5‐(CF_3_)_2_C_6_H_3_	**8e**	75
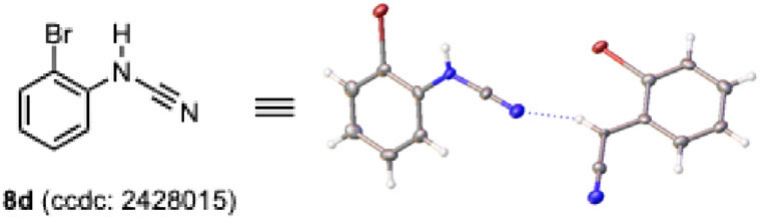			

^[a]^ Isolated yield.

**Scheme 3:**
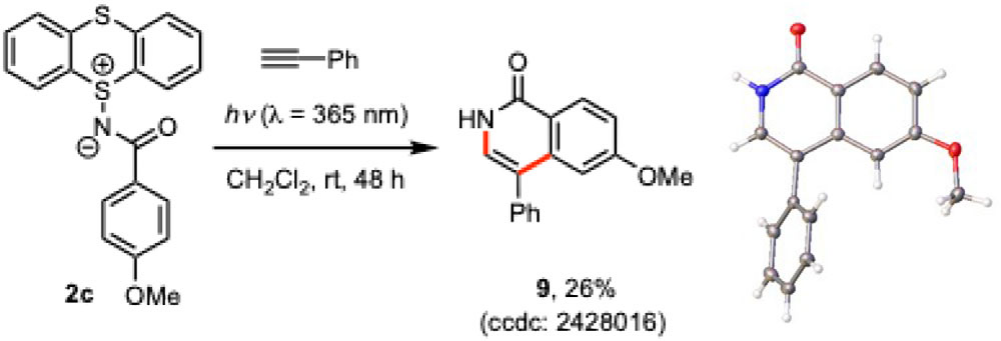
Utilization of *N*‐acyliminothianthrene **2c** as an *N*‐acylnitrene equivalent.

Finally, the potential of *N*‐acyliminothianthrenes as *N*‐acylnitrene equivalents was briefly investigated (Scheme 3 and Scheme S1). Although the reaction conditions have not yet been fully optimized, **2c** reacted with phenylacetylene under photo‐irradiation, followed by subsequent cyclization to afford isoquinolinone **9**.^[^ ^33^, ^34^ ^]^

## 3 Conclusion

We have demonstrated that 5‐iminothianthrene reacts with various electrophiles, such as acid chlorides, protons, and isothiocyanates, under mild conditions. It was found that the obtained adducts exhibit two types of intramolecular ChB: (1) σ* orbital of the C─S bond in the thianthrene scaffold interacts with the lone pair of the (thio)carbonyl group, regulating the conformation; and (2) the σ* orbital of the S─N bond in *N*‐acyliminothianthrene interacts with the lone pair of the other sulfur atom, and this interaction is likely responsible for the stability. In the case of the isothiocyanate adduct, a relatively strong interaction (ca. 5.3 kcal/mol) was identified, which unexpectedly triggered a metathesis‐type reaction, leading to the formation of cyanamide. While *N*‐acyliminothianthrene can be easily isolated and handled, photo‐irradiation enables its use in electrophilic amination. Further optimization and application of the adducts are currently under investigation in our laboratory.

## Supporting Information

The detailed experimental procedures, compound characterization data, copies of NMR spectra, and computational studies are provided in the Supporting Information. Deposition Numbers 2427580 (for **2a**), 2427626 (for **2b**), 2428989 (for **2c**), 2427543 (for **2d**), 2427632 (for **2e**), 2428009 (for **3**), 2428010 (for **4**), 2428012 (for **5**), 2428013 (for **6a**), 2428014 (for **7a**), 2428015 (for **8d**), and 2428016 (for **9**) contains the supplementary crystallographic data for this paper. These data are provided free of charge by the joint Cambridge Crystallographic Data Centre and Fachinformationszentrum Karlsruhe Access Structures service.

## Conflict of Interests

The authors declare no conflict of interest.

## Supporting information



Supporting Information

Supporting Information

## Data Availability

The data that support the findings of this study are available in the supplementary material of this article.
