# Supplementary material for: Impacts of Chalcogen Bonding on the Stability and Reactivity of 5‐Iminothianthrene Platform: Toward Electrophilic Nitrogen Sources
Source: Chemistry. 2025 May 13;31(33):e202501045. doi: 10.1002/chem.202501045 (PMC12160997; doi:10.1002/chem.202501045)

Supporting Information

for

Impacts of Chalcogen Bonding on the Stability and Reactivity of 5-Iminothianthrene Platform: Toward Electrophilic Nitrogen Sources

Ryutaro Tawara,[a] Shohei Hamada,[b] Takumi Furuta,[a]*and Yusuke Kobayashi[a]*

[a] *Department of Pharmaceutical Chemistry, Kyoto Pharmaceutical University, Yamashina-ku, Kyoto 607-8414, Japan*

[b] *Division of Pharmaceutical Sciences, Graduate School of Medical Sciences, Kanazawa University, Kanazawa 920-0017, Japan*

CONTENTS

1. General information--- S3

2. General procedure for the synthesis of *N*-acyliminothianthrene (2) ------------------------- S3

3. Crystal data and structure refinement for N-acyliminothianthrene 2a-e ------------------- S4

4. DFT calculation -- S7

5. Procedure for the protonation of 1 and 2a-- S20

6. Reaction of 1 with iso(thio)cyanate --- S23

7. General procedure for the synthesis of cyanamide from 7 ---------------------------------- S27

8. Reaction of 2c with phenylacetylene under photo-irradiation ----------------------------- S28

9. References -- S30

10. Copies of ^{1}H and ^{13}C NMR charts --- S31

1. General information

Unless otherwise noted, all chemicals and solvents were obtained from commercial suppliers and used without further purification. Column chromatography on silica gel was carried out using CHROMATOREX PSQ100B (spherical, neutral, 100 µm, Fuji-silysia Co., Inc.). TLC analysis and preparative TLC analysis were performed on commercial glass plates bearing a 0.25 mm layer or 0.5 mm layer of Merck Kiesel-gel 60 F254, respectively. All melting points were measured by using a Büchi Melting Point M-565, and are uncorrected. NMR spectra were obtained with a Bruker UltraShield 300, or a Bruker Ascend 500 spectrometer. Chemical shifts are given in units of ppm (^{1}H NMR in $CDCl_3$: tetramethylsilane as the internal standard at 0 ppm, and $CDCl_3$ as the internal standard at 7.26 ppm; ^{13}C NMR in $CDCl_3$: $CDCl_3$ as the internal standard at 77.0 ppm; ^{1}H NMR in DMSO-d_6: DMSO-d_6 as the internal standard at 2.50 ppm; ^{13}C NMR in DMSO-d_6: DMSO-d_6 as the internal standard at 39.5 ppm). Spin-spin coupling constants are in Hz. HRMS was recorded on a JEOL GCmate II (for EI), a JEOL MStation JMS-700 spectrometer (for FAB). X-ray crystallographic analyses were performed using an XtaLAB Synergy-DW (Rigaku Co., Inc).

2. General procedure for the synthesis of *N*-acyliminothianthrene (2)

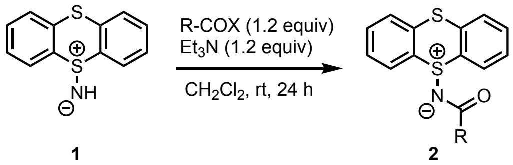

To a solution of 5-iminothianthrene[23] (150.8 mg, 0.652 mmol) (**1**) and Et_3N (109 µL, 0.782 mmol) in CH_2Cl_2 (6.5 mL), was added acetyl chloride (55.6 µL, 0.782 mmol) dropwise at room temperature, and the reaction mixture was stirred at the same temperature for 24 h. The resulting mixture was poured into water, and extracted with CH_2Cl_2 three times. The combined organic layer was dried over anhydrous Na_2SO_4 and concentrated under reduced pressure. The crude product was purified by recrystallization using hexane. *N*-(5λ^4-thianthren-5-ylidene)acetamide (**2a**) was obtained as a white solid (156.9 mg, 88%).

_N_-(5λ^4-thianthren-5-ylidene)acetamide(2a) [27]

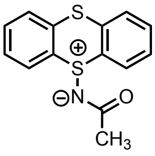

2a

^{1}H NMR (500 MHz, $CDCl_3$) δ 7.92 (d, J = 8.9 Hz, 2H), 7.69 (d, J = 7.6 Hz, 2H), 7.53 (t, J = 7.5 Hz, 2H), 7.49 (td, J = 7.3, 1.4 Hz, 2H), 2.37 (s, 3H). The spectral data were consistent with the literature data.[27]

_N_-(5λ^4-thianthren-5-ylidene)benzamide (2b) [27]

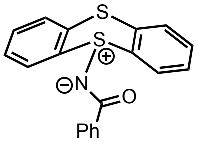

Yield: 71%; ^{1}H NMR (500 MHz, $CDCl_3$) δ 8.39 (d, J = 7.6 Hz, 2H), 8.02 – 7.96 (m, 2H), 7.71 (d, J = 7.5 Hz, 2H), 7.57 – 7.46 (m, 8H). The spectral data were consistent with the literature data.[27]

_N_-(5λ⁴-thianthren-5-ylidene)-4-methoxybenzamide (2c) [35]

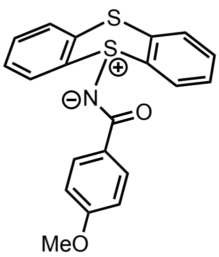

Yield: 94%; ^{1}H NMR (500 MHz, $CDCl_3$) δ 8.36 (d, *J* = 8.7 Hz, 2H), 7.98 (d, *J* = 7.9 Hz, 2H), 7.70 (d, *J* = 7.6 Hz, 2H), 7.54 (t, *J* = 7.6 Hz, 2H), 7.49 (t, *J* = 7.5 Hz, 2H), 6.98 (d, *J* = 8.9 Hz, 2H), 3.89 (s, 3H). The spectral data were consistent with the literature data.[35]

_N_-(5λ⁴-thianthren-5-ylidene)-2,2,2-trifluoroacetamide (2d)

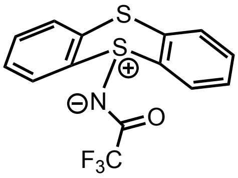

Yield: 84%; M.p. 125.0-127.9 ˚C (CH_2Cl_2/*n*-hexane); ^{1}H NMR (500 MHz, $CDCl_3$) δ 7.91 (dd, *J* = 7.5, 1.9 Hz, 2H), 7.75 (dd, *J* = 6.8, 2.3 Hz, 2H), 7.57 (tt, *J* = 7.4, 5.7 Hz, 4H); ^{13}C NMR (75 MHz, $CDCl_3$) δ 168.87 (q, *J* = 35.7 Hz), 132.59, 131.79, 130.08, 129.94, 129.07, 127.87, 117.19 (q, *J* = 288.2 Hz); ^{19}F NMR (282 MHz, $CDCl_3$) δ –73.24; HRMS (EI): *m*/*z* calcd for $C_{14}H_8F_3NOS_2$ $[M]^+$ 326.9999, found 326.9993.

***tert*-Butyl (5λ⁴-thianthren-5-ylidene)carbamate (2e)**

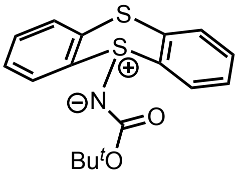

Yield: 75%; M.p. 165.2-172.7 ˚C (CH_2Cl_2/*n*-hexane); ^{1}H NMR (500 MHz, $CDCl_3$) δ 7.94 (d, *J* = 7.9 Hz, 2H), 7.66 (d, *J* = 7.6 Hz, 2H), 7.53 (t, *J* = 7.6 Hz, 2H), 7.47 (t, *J* = 7.2 Hz, 2H), 1.58 (s, 10H); ^{13}C NMR (75 MHz, $CDCl_3$) δ ^{13}C NMR (75 MHz, $CDCl_3$) δ 165.48, 134.07, 131.27, 130.66, 129.49, 128.71, 126.46, 79.91, 77.36, 28.60; HRMS (EI): *m*/*z* calcd for $C_{17}H_{17}NO_2S_2$ $[M]^+$ 331.0701, found 331.0693.

3. Crystal data and structure refinement for *N*-acyliminothianthrene 2a-e

Single crystals of **2a-e** were obtained by recrystallization from CH_2Cl_2/*n*-hexane. A suitable crystal was selected and loop on a XtaLAB AFC11 (RCD3): quarter-chi single diffractometer. The crystal was kept at 100 K during data collection. Using Olex[36] the structure was solved with the ShelXT[37] structure solution program using Intrinsic Phasing and refined with the ShelXL[38] refinement package using Least Squares minimization.

Table S1. Summary of the crystal data of 2a-c

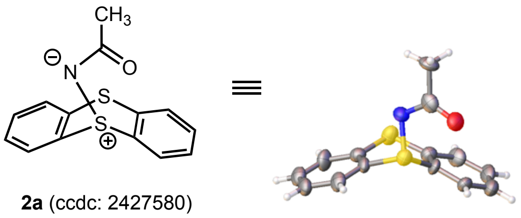

2a (ccdc: 2427580)

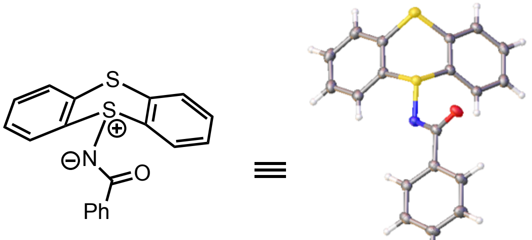

2b (ccdc: 2427626)

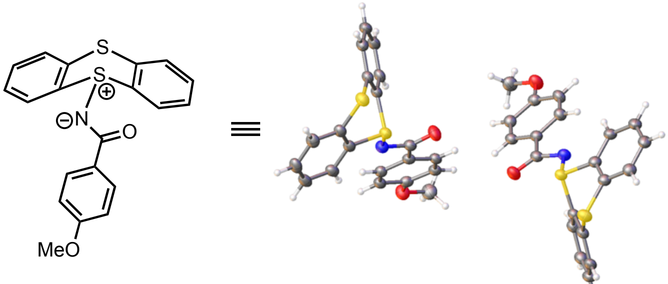

2c (ccdc: 2428989)

	2a	2b	2c
CCDC	2427580	2427626	2428989
Empirical formula	$C_{14}H_{11}NOS_2$	$C_{19}H_{13}NOS_2$	$C_{40}H_{30}N_2O_4S_4$
Formula weight	273.36	335.42	730.90
Temperature/K	296.15	100.00(10)	99.8(4)
Crystal system	Monoclinic	Triclinic	Triclinic
Space group	$P2_1$	P-1	P-1
a/Å	10.0085(12)	8.5856(3)	8.1085(2)
b/Å	5.1332(6)	10.5791(4)	11.2201(2)
c/Å	12.0659(14)	10.6474(3)	19.0042(3)
α/°	90	104.541(3)	86.7680(10)
β/°	91.055(6)	108.373(3)	87.546(2)
γ/°	90	111.912(3)	84.853(2)
Volume/Å^3	619.78(13)	773.23(5)	1718.02(6)
Z	2	2	2
ρ_{calc}g/cm^3	1.465	1.441	1.413
μ/mm^{-1}	3.770	3.138	2.917
F(000)	284.0	348.0	760.0
Crystal size/mm^3	0.5 × 0.5 × 0.2	0.2 × 0.2 × 0.1	0.2 × 0.2 × 0.1
Radiation	Cu Kα (λ = 01.54187)	Cu Kα (λ = 1.54184)	Cu Kα (λ = 1.54184)
2Θ range for data collection/°	7.328 to 136.294	9.638 to 150.2	4.66 to 156.522
Index (h, k, lmax)	11, 5, 13	8, 13, 13	9, 14, 23
Reflections collected	6974	8933	32340
Independent reflections	2042 [R_{int} = 0.1021, R_{sigma} = 0.1430]	3066 [R_{int} = 0.0263, R_{sigma} = 0.0283]	7069 [R_{int} = 0.0468, R_{sigma} = 0.0243]
Goodness-of-fit on F^2	1.054	1.007	1.045
Final R indexes [I>=2σ (I)]	R_1 = 0.0807, wR_2 = 0.1246	R_1 = 0.0404, wR_2 = 0.1122	R_1 = 0.0467, wR_2 = 0.1308
Final R indexes [all data]	R_1 = 0.1396, wR_2 = 0.1580	R_1 = 0.0429, wR_2 = 0.1145	R_1 = 0.0500, wR_2 = 0.1333

Table S2. Summary of the crystal data of 2d and 2e

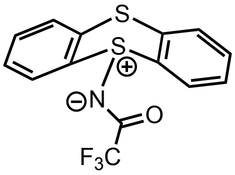

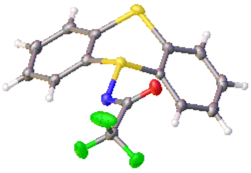

2d (ccdc: 2427543)

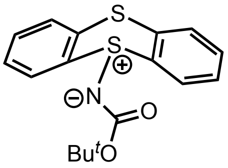

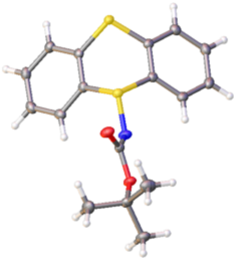

2e (ccdc: 2427632)

	**2d**	**2e**
CCDC	2427543	2427632
Empirical formula	$C_{14}H_8F_3NOS_2$	$C_{17}H_{17}NO_2S_2$
Formula weight	327.33	331.43
Temperature/K	100.00(10)	100.00(10)
Crystal system	Orthorhombic	Triclinic
Space group	$P2_12_12_1$	P-1
a/Å	4.2713(2)	9.1261(4)
b/Å	11.4788(5)	9.2832(4)
c/Å	26.7771(11)	9.8793(4)
α/°	90	87.616(3)
β/°	90	73.924(4)
γ/°	90	81.123(3)
Volume/Å^3	1312.86(10)	794.61(6)
Z	4	2
ρ_{calc}g/cm^3	1.656	1.385
μ/mm^{-1}	4.014	3.086
F(000)	664.0	348.0
Crystal size/mm^3	0.3 × 0.05 × 0.05	0.3 × 0.2 × 0.2
Radiation	Cu Kα (λ = 1.54184)	Cu Kα (λ = 1.54184)
2Θ range for data collection/°	6.6.02 to 150.042	9.316 to 151.382
Index (h, k, lmax)	5, 14, 25	11, 11, 10
Reflections collected	7483	15110
Independent reflections	2530 [R_{int} = 0.0445, R_{sigma} = 0.0378]	3185 [R_{int} = 0.0416, R_{sigma} = 0.0294]
Goodness-of-fit on F^2	1.074	1.072
Final R indexes [I>=2σ (I)]	R_1 = 0.0438, wR_2 = 0.1121	R_1 = 0.0317, wR_2 = 0.0851
Final R indexes [all data]	R_1 = 0.0447, wR_2 = 0.1128	R_1 = 0.0350, wR_2 = 0.0869

4. DFT calculation

Based on the X-ray structure, DFT calculation, including the natural bond orbital (NBO) analyses were performed at the ωB97XD/6-31+G(d,p) level[39] using Gaussian 16 software package.[40] The molecular geometries for each transition states were first estimated with the Reaction plus software package, based on the nudged elastic band method,[41] and were subsequently re-optimized using the Gaussian program. Once the stationary points were obtained at ωB97XD/6-31G(d,p) level, the harmonic vibrational frequencies were calculated at the same level to estimate the Gibbs free energy. The nature of the stationary points was characterized via vibrational analysis. All of the Gibbs free energy values reported in this paper were calculated for a temperature of 298.15 K. The transition structure reported was optimized without constraints and the intrinsic reaction coordinate (IRC) route was calculated in both directions toward the corresponding minima for each transition-state structure.

4-1. Axial/equatorial interconversion of 1

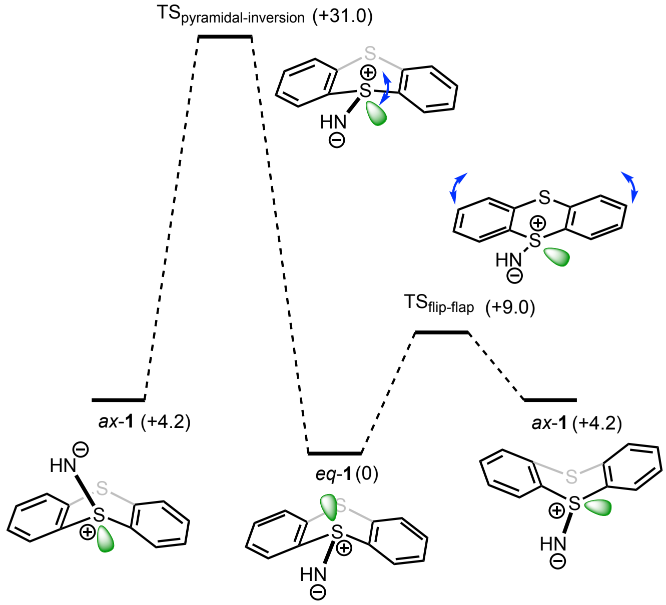

Figure 1B. Axial-equatorial isomerization of **1**.Values in parentheses are given in kcal/mol.

ax-1

0 1

S	-1.67195300	0.36861600	0.00000000
S	1.59245800	0.98484200	0.00000000
N	-1.95639600	1.92034900	0.00000000
C	0.78869200	0.19045500	1.37109600
C	-1.15523000	-0.76908600	2.43981300
H	-2.21196900	-1.01686300	2.40589900
C	-1.15523000	-0.76908600	-2.43981300
H	-2.21196900	-1.01686300	-2.40589900
C	0.78869200	0.19045500	-1.37109600

C	-0.56897800	-0.13825600	1.34466700
C	-0.56897800	-0.13825600	-1.34466700
C	1.54515300	-0.09499700	-2.50835600
H	2.60317100	0.14617900	-2.52404300
C	-0.39946500	-1.05481600	-3.57047700
H	-0.86053600	-1.54190100	-4.42283100
C	1.54515300	-0.09499700	2.50835600
H	2.60317100	0.14617900	2.52404300
C	0.94800900	-0.70623200	-3.60491500
H	1.54476800	-0.92561300	-4.48409800
C	-0.39946500	-1.05481600	3.57047700
H	-0.86053600	-1.54190100	4.42283100
C	0.94800900	-0.70623200	3.60491500
H	1.54476800	-0.92561300	4.48409800
H	-1.08235700	2.45380800	0.00000000

Calculation Type = FREQ

Calculation Method = RwB97XD

Formula = C12H9NS2

Basis Set = 6-31G(d,p)

Charge = 0

Spin = Singlet

Solvation = None

Imaginary Freq = 0

Temperature = 298.15 Kelvin

Pressure = 1 atm

Frequencies scaled by = 1

Electronic Energy (EE) = -1313.5436 Hartree

Zero-point Energy Correction = 0.180123 Hartree

Thermal Correction to Energy = 0.19199 Hartree

Thermal Correction to Enthalpy = 0.192934 Hartree

Thermal Correction to Free Energy = 0.141844 Hartree

EE + Zero-point Energy = -1313.3635 Hartree

EE + Thermal Energy Correction = -1313.3516 Hartree

EE + Thermal Enthalpy Correction = -1313.3507 Hartree

EE + Thermal Free Energy Correction = -1313.4018 Hartree

Maximum displacement = 0.001145 Converged

RMS displacement = 0.00015 Converged

eq-1

0 1

S	0.00000000	1.42240100	-0.90172700
S	0.00000000	-1.77546500	-1.01917600
N	0.00000000	2.87321800	-0.24540700
C	1.35591300	-0.90882300	-0.25187700
C	2.36408900	1.17038700	0.48649200
H	2.27891900	2.25030000	0.57016400
C	-2.36408900	1.17038700	0.48649200
H	-2.27891900	2.25030000	0.57016400
C	-1.35591300	-0.90882300	-0.25187700
C	1.34162200	0.48109100	-0.14711800
C	-1.34162200	0.48109100	-0.14711800
C	-2.44222800	-1.61519400	0.26189300
H	-2.45616100	-2.69836800	0.20043200
C	-3.44824900	0.45913000	0.99059400
H	-4.25874000	0.98657700	1.48230900
C	2.44222800	-1.61519400	0.26189300
H	2.45616100	-2.69836800	0.20043200
C	-3.49085300	-0.92727200	0.86565800
H	-4.33604400	-1.48248700	1.25899000
C	3.44824900	0.45913000	0.99059400
H	4.25874000	0.98657700	1.48230900
C	3.49085300	-0.92727200	0.86565800
H	4.33604500	-1.48248700	1.25899000
H	0.00000000	3.51262900	-1.03921400

Calculation Type = FREQ

Calculation Method = RwB97XD

Formula = C12H9NS2

Basis Set = 6-31G(d,p)

Charge = 0

Spin = Singlet

Solvation = None

Imaginary Freq = 0

Temperature = 298.15 Kelvin

Pressure = 1 atm

Frequencies scaled by = 1

Electronic Energy (EE) = -1313.5509 Hartree

Zero-point Energy Correction = 0.180252 Hartree

Thermal Correction to Energy = 0.192029 Hartree

Thermal Correction to Enthalpy = 0.192973 Hartree

Thermal Correction to Free Energy = 0.14239 Hartree

EE + Zero-point Energy = -1313.3706 Hartree

EE + Thermal Energy Correction = -1313.3588 Hartree

EE + Thermal Enthalpy Correction = -1313.3579 Hartree

EE + Thermal Free Energy Correction = -1313.4085 Hartree

TS$_{\text{pyramidal-inversion}}$

0 1

S	-0.00002000	1.48552000	0.30632700
S	0.00004200	-1.69453700	0.93162400
N	-0.00020200	3.04305200	0.74334800
C	-1.39286500	-0.88542400	0.17628800
C	-2.58115800	1.13056000	-0.47753300
H	-2.57682200	2.20759700	-0.60852600
C	2.58118700	1.13063100	-0.47742400
H	2.57683800	2.20768000	-0.60831700
C	1.39290200	-0.88540100	0.17624500
C	-1.43328900	0.50011400	-0.00355200
C	1.43331000	0.50013700	-0.00352300
C	2.51981600	-1.64005800	-0.15207000
H	2.48674900	-2.71857700	-0.03692200
C	3.71163000	0.37086600	-0.74969800
H	4.61432000	0.86032700	-1.09903300
C	-2.51977200	-1.64010500	-0.15198000
H	-2.48670000	-2.71861700	-0.03676900
C	3.67836900	-1.01318700	-0.59651100
H	4.55494800	-1.60875800	-0.82752100
C	-3.71159400	0.37076700	-0.74977500
H	-4.61428000	0.86019600	-1.09916300
C	-3.67832700	-1.01326900	-0.59647700
H	-4.55490000	-1.60886200	-0.82744900
H	-0.00033300	3.00813000	1.76910500

Calculation Type = FREQ

Calculation Method = RwB97XD

Formula = C12H9NS2

Basis Set = 6-31G(d,p)

Charge = 0

Spin = Singlet

Solvation = None

Imaginary Freq = 1 (–403.44 cm^{-1})

Temperature = 298.15 Kelvin

Pressure = 1 atm

Frequencies scaled by = 1

Electronic Energy (EE) = -1313.5002 Hartree

Zero-point Energy Correction = 0.17922 Hartree

Thermal Correction to Energy = 0.190757 Hartree

Thermal Correction to Enthalpy = 0.191701 Hartree

Thermal Correction to Free Energy = 0.141114 Hartree

EE + Zero-point Energy = -1313.321 Hartree

EE + Thermal Energy Correction = -1313.3094 Hartree

EE + Thermal Enthalpy Correction = -1313.3085 Hartree

EE + Thermal Free Energy Correction = -1313.3591 Hartree

TS$_{\text{flip-flap}}$

0 1

S	0.00000500	1.63736700	-0.22031500
S	0.00000800	-1.93141200	0.19593200
N	-0.00001900	2.62233300	1.01454600
C	-1.41239500	-0.88984700	0.01555900
C	-2.62744100	1.17295200	-0.27969500
H	-2.60964300	2.25321000	-0.39132000
C	2.62743000	1.17295100	-0.27976200
H	2.60962600	2.25319900	-0.39148500
C	1.41240800	-0.88983900	0.01563600
C	-1.41012900	0.49200500	-0.16588300
C	1.41012500	0.49201200	-0.16584100
C	2.63846400	-1.56970700	0.07021100
H	2.64234500	-2.64674200	0.21013900
C	3.83487400	0.49788500	-0.22476500
H	4.76764700	1.04291300	-0.31719300

C	-2.63844800	-1.56972800	0.07010500
H	-2.64231500	-2.64677200	0.20996300
C	3.83545600	-0.88491700	-0.04871800
H	4.77116000	-1.43209100	-0.00336000
C	-3.83487900	0.49787700	-0.22469200
H	-4.76765800	1.04291000	-0.31702600
C	-3.83544700	-0.88494100	-0.04875700
H	-4.77114400	-1.43212700	-0.00343100
H	-0.00019300	2.09367600	1.89161300

Calculation Type = FREQ

Calculation Method = RwB97XD

Formula = C12H9NS2

Basis Set = 6-31G(d,p)

Charge = 0

Spin = Singlet

Solvation = None

Imaginary Freq = 1

Temperature = 298.15 Kelvin

Pressure = 1 atm

Frequencies scaled by = 1 (–54.99 cm^{-1})

Electronic Energy (EE) = -1313.5366 Hartree

Zero-point Energy Correction = 0.179686 Hartree

Thermal Correction to Energy = 0.190903 Hartree

Thermal Correction to Enthalpy = 0.191847 Hartree

Thermal Correction to Free Energy = 0.142431 Hartree

EE + Zero-point Energy = -1313.3569 Hartree

EE + Thermal Energy Correction = -1313.3457 Hartree

EE + Thermal Enthalpy Correction = -1313.3448 Hartree

EE + Thermal Free Energy Correction = -1313.3942 Hartree

4-2. Axial/equatorial interconversion of 2b

The activation energy for the transition state (TS) of flip-flap isomerization of **2b** is 7.1 kcal/mol, whereas the activation energy for the TS of pyramidal inversion exceeds 30 kcal/mol (Figure S1). These results suggest that the axial form of **2** can be obtained as the kinetic product.

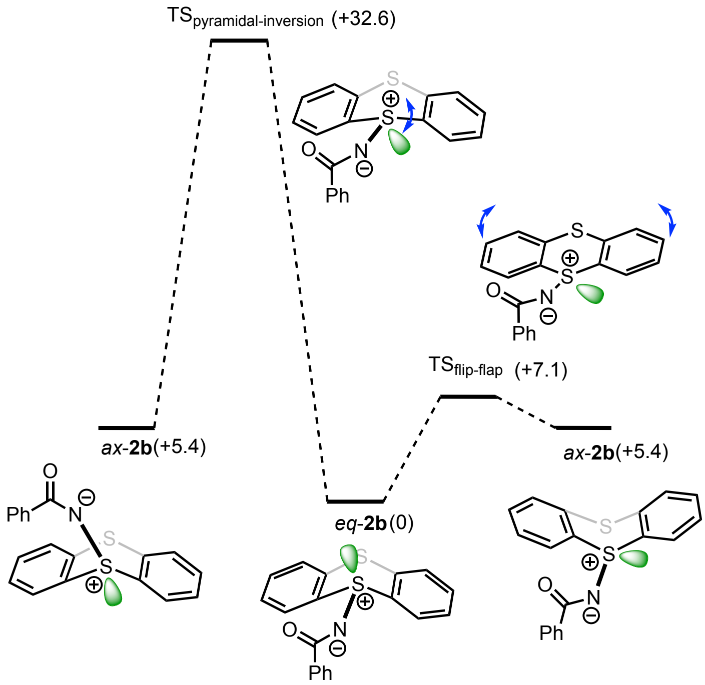

Figure S1. Axial/equatorial interconversion of **2b**. Values in parentheses are given in kcal/mol.

ax-2b

0 1

S	-0.62287100	-0.00010200	-1.49228500
S	-2.34459200	-0.00009000	1.57221900
O	0.70453700	0.00087100	1.02516800
N	0.98413400	0.00007900	-1.27531700
C	1.42207500	0.00044800	0.02696300
C	-2.05174200	1.39866700	0.55070000
C	-1.25329700	2.59081400	-1.40217400
H	-0.74336500	2.56903400	-2.36068700
C	-1.25297100	-2.59109300	-1.40195800
H	-0.74303200	-2.56932700	-2.36046800
C	-2.05159100	-1.39889100	0.55080400
C	-1.39780400	1.40094300	-0.68238400
C	-1.39762600	-1.40118300	-0.68226400
C	-2.54032500	-2.61387800	1.04981700
H	-3.05816500	-2.62331000	2.00398900

C	-1.72860200	-3.78794400	-0.89283600
H	-1.60317000	-4.70658100	-1.45456100
C	-2.54062800	2.61363500	1.04961500
H	-3.05846500	2.62308100	2.00378800
C	-2.37052500	-3.79349900	0.34407600
H	-2.75212000	-4.72121700	0.75719000
C	-1.72907400	3.78764600	-0.89314500
H	-1.60376500	4.70625200	-1.45494700
C	-2.37098600	3.79321900	0.34377300
H	-2.75269900	4.72092200	0.75681400
C	2.91805600	0.00024700	0.15710800
C	3.76216600	-0.00022600	-0.95513900
C	3.46616300	0.00056100	1.44054000
C	5.14184000	-0.00038300	-0.78136800
H	3.32466000	-0.00046300	-1.94692800
C	4.84501100	0.00040600	1.61253700
H	2.78982500	0.00092800	2.28834500
C	5.68485100	-0.00006800	0.50117200
H	5.79552600	-0.00075100	-1.64811600
H	5.26649900	0.00065300	2.61294500
H	6.76250300	-0.00019100	0.63454000

Calculation Type = FREQ

Calculation Method = RwB97XD

Formula = C19H13NOS2

Basis Set = 6-31G(d,p)

Charge = 0

Spin = Singlet

Solvation = None

Imaginary Freq = 0

Temperature = 298.15 Kelvin

Pressure = 1 atm

Frequencies scaled by = 1

Electronic Energy (EE) = -1657.8441 Hartree

Zero-point Energy Correction = 0.272537 Hartree

Thermal Correction to Energy = 0.290751 Hartree

Thermal Correction to Enthalpy = 0.291695 Hartree

Thermal Correction to Free Energy = 0.224529 Hartree

EE + Zero-point Energy = -1657.5716 Hartree

EE + Thermal Energy Correction = -1657.5534 Hartree

EE + Thermal Enthalpy Correction = -1657.5524 Hartree

EE + Thermal Free Energy Correction = -1657.6196 Hartree

E (Thermal) = 182.449 kcal/mol

eq-2b

0 1

S	-0.41955800	-0.04546200	0.81734100
S	-3.55839100	0.21834500	0.96543200
O	1.79018000	1.32925800	1.43969300
N	1.01080500	-0.37053100	0.08825600
C	1.99055200	0.39582200	0.65600300
C	-2.61229100	1.43449600	0.07101300
C	-0.47610200	2.23690400	-0.75836600
H	0.59746800	2.11418900	-0.83456600
C	-0.84721800	-2.52595000	-0.33974500
H	0.23163300	-2.56264700	-0.43496900
C	-2.81741800	-1.26269700	0.31028300
C	-1.22886900	1.31345000	-0.04689200
C	-1.43230900	-1.38133600	0.18603000
C	-3.62538200	-2.32730200	-0.08599400
H	-4.70372400	-2.23621300	-0.01180600
C	-1.66374700	-3.58558600	-0.71992300
H	-1.21710000	-4.48763900	-1.12372000
C	-3.25068600	2.52123100	-0.52385800
H	-4.32932800	2.61360000	-0.45484700
C	-3.04585700	-3.48915800	-0.58451400
H	-3.68065000	-4.31618700	-0.88446200
C	-1.12154500	3.32398100	-1.33781900
H	-0.54354500	4.06041500	-1.88506300
C	-2.50054700	3.46871000	-1.21266600
H	-3.00067200	4.31708400	-1.66762800
C	3.38083700	0.03900700	0.21874800
C	3.63318400	-0.96401400	-0.71929700
C	4.44636900	0.74585900	0.77736500
C	4.94028800	-1.25749700	-1.09092900
H	2.79799400	-1.50108200	-1.15469500

C	5.75281000	0.44898300	0.40791000
H	4.22443900	1.52294500	1.50061700
C	6.00140500	-0.55312500	-0.52690500
H	5.13260900	-2.03657400	-1.82203100
H	6.57840500	0.99946300	0.84793600
H	7.02174200	-0.78451500	-0.81713700

Calculation Type = FREQ

Calculation Method = RwB97XD

Formula = C19H13NOS2

Basis Set = 6-31G(d,p)

Charge = 0

Spin = Singlet

Solvation = None

Imaginary Freq = 0

Temperature = 298.15 Kelvin

Pressure = 1 atm

Frequencies scaled by = 1

Electronic Energy (EE) = -1657.8526 Hartree

Zero-point Energy Correction = 0.272484 Hartree

Thermal Correction to Energy = 0.290735 Hartree

Thermal Correction to Enthalpy = 0.29168 Hartree

Thermal Correction to Free Energy = 0.22437 Hartree

EE + Zero-point Energy = -1657.5801 Hartree

EE + Thermal Energy Correction = -1657.5618 Hartree

EE + Thermal Enthalpy Correction = -1657.5609 Hartree

EE + Thermal Free Energy Correction = -1657.6282 Hartree

TS$_{\text{pyramidal-inversion}}$

0 1

S	0.38754700	0.00039300	-0.46834000
S	3.02550600	-0.00068000	1.35772900
O	-1.44974800	-0.00034700	1.59314500
N	-1.22602800	0.00062800	-0.74001600
C	-1.92662800	0.00022200	0.46989500
C	2.57560000	-1.39780700	0.35242200
C	0.98789700	-2.58983600	-1.05816800
H	0.05775100	-2.58518500	-1.61593800

C	0.98981100	2.59043700	-1.05770700
H	0.05952000	2.58678300	-1.61522200
C	2.57682300	1.39669600	0.35221900
C	1.38107600	-1.44506800	-0.37144000
C	1.38224200	1.44509500	-0.37151500
C	3.39471100	2.52705700	0.36001600
H	4.33608600	2.49500200	0.89856000
C	1.79081800	3.72327300	-0.99080400
H	1.48076800	4.62837700	-1.50127400
C	3.39253900	-2.52885500	0.36011600
H	4.33386400	-2.49771600	0.89880100
C	2.99437200	3.68844500	-0.29106100
H	3.62875400	4.56735600	-0.25518600
C	1.78791800	-3.72337300	-0.99137800
H	1.47729700	-4.62804600	-1.50226500
C	2.99132400	-3.68974900	-0.29130800
H	3.62491800	-4.56923000	-0.25545700
C	-3.41387900	0.00055700	0.25259400
C	-3.98914700	0.00045500	-1.02042900
C	-4.23478700	0.00072200	1.38194000
C	-5.37296400	0.00054800	-1.15770300
H	-3.34386600	0.00033000	-1.89105300
C	-5.61703100	0.00083000	1.24209900
H	-3.76487000	0.00072800	2.35929500
C	-6.18792100	0.00073500	-0.02849900
H	-5.81694400	0.00047500	-2.14828800
H	-6.25044900	0.00098800	2.12356800
H	-7.26813900	0.00080000	-0.13853700

Calculation Type = FREQ

Calculation Method = RwB97XD

Formula = C19H13NOS2

Basis Set = 6-31G(d,p)

Charge = 0

Spin = Singlet

Solvation = None

Imaginary Freq = 1 (–322.63 cm^{-1})

Temperature = 298.15 Kelvin

Pressure = 1 atm

Frequencies scaled by = 1

Electronic Energy (EE) = -1657.7988 Hartree

Zero-point Energy Correction = 0.27122 Hartree

Thermal Correction to Energy = 0.289229 Hartree

Thermal Correction to Enthalpy = 0.290173 Hartree

Thermal Correction to Free Energy = 0.222545 Hartree

EE + Zero-point Energy = -1657.5276 Hartree

EE + Thermal Energy Correction = -1657.5096 Hartree

EE + Thermal Enthalpy Correction = -1657.5087 Hartree

EE + Thermal Free Energy Correction = -1657.5763 Hartree

TS$_{\text{flip-flap}}$

0 1

S	0.61069900	0.00003400	-1.50772200
S	3.02701000	-0.00027500	1.08151900
N	-0.99672300	0.00006200	-1.29428900
C	2.26342300	-1.41325100	0.36006700
C	0.80197100	-2.63265700	-1.13423900
H	0.04302400	-2.61478500	-1.91038800
C	0.80258200	2.63268000	-1.13416000
H	0.04360400	2.61501200	-1.91028400
C	2.26378800	1.41290100	0.36007700
C	1.31326300	-1.42395900	-0.65682600
C	1.31358700	1.42385000	-0.65677500
C	2.69502500	2.63830000	0.88489800
H	3.42916100	2.64195300	1.68490600
C	1.23291700	3.83845100	-0.60527400
H	0.82652500	4.77092000	-0.98011900
C	2.69432000	-2.63876200	0.88490800
H	3.42841400	-2.64260700	1.68495300
C	2.18730800	3.83594400	0.40905000
H	2.53539400	4.77031900	0.83617800
C	1.23198500	-3.83853900	-0.60534500
H	0.82538100	-4.77090400	-0.98021500
C	2.18632800	-3.83627500	0.40902700
H	2.53415600	-4.77073900	0.83617000
C	-1.39587300	0.00010600	0.02681100

O	-0.64369300	0.00007400	0.99868100
C	-2.88619000	0.00018600	0.20252500
C	-3.76214400	0.00019300	-0.88476300
C	-3.39609700	0.00025700	1.50159900
C	-5.13611000	0.00026900	-0.67087600
H	-3.35311200	0.00014100	-1.88872200
C	-4.76938000	0.00033500	1.71358700
H	-2.69501200	0.00024900	2.32913200
C	-5.64124900	0.00034000	0.62711300
H	-5.81492200	0.00027300	-1.51806700
H	-5.16155400	0.00039100	2.72582500
H	-6.71450400	0.00040100	0.79203000

Calculation Type = FREQ

Calculation Method = RwB97XD

Formula = C19H13NOS2

Basis Set = 6-31G(d,p)

Charge = 0

Spin = Singlet

Solvation = None

Imaginary Freq = 1 (–35.37 cm^{-1})

Temperature = 298.15 Kelvin

Pressure = 1 atm

Frequencies scaled by = 1

Electronic Energy (EE) = -1657.8426 Hartree

Zero-point Energy Correction = 0.272274 Hartree

Thermal Correction to Energy = 0.289699 Hartree

Thermal Correction to Enthalpy = 0.290643 Hartree

Thermal Correction to Free Energy = 0.225762 Hartree

EE + Zero-point Energy = -1657.5703 Hartree

EE + Thermal Energy Correction = -1657.5529 Hartree

EE + Thermal Enthalpy Correction = -1657.552 Hartree

EE + Thermal Free Energy Correction = -1657.6168 Hartree

4-3. NBO analysis of 2a (Table S3: based on the X-ray structure)

Intramolecular ChB was identified between the lone pair of carbonyl oxygen atom and the σ* orbital of S-C bond with interaction energies of 2.64 kcal/mol (entry 1). In addition, intermolecular ChB ($LP_S \rightarrow \sigma^*_{S\text{–}N}$) was also observed in the axial form of **2a** (entry 2), which likely contributes to its good crystallinity.

Table S3. Second order perturbation theory analysis of Fock matrix in NBO basis

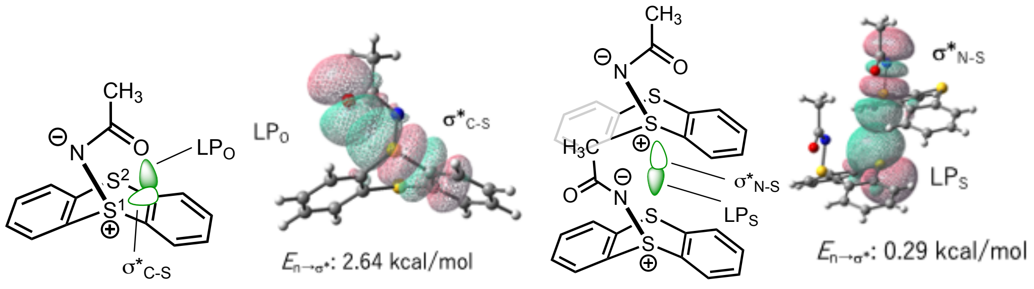

entry	Donor NBO of **2a** (i)	Acceptor NBO of **2a** (j)	E(2) kcal/mol	E(j) – E(i) a.u.	F(i,j) a.u.
1	LP O3<br>(Orbital#70)	BD* S1–C12<br>(Orbital#80)	2.64	0.56	0.035
2	LP S31<br>(Orbital#130)	BD*(1) S1–N4<br>(Orbital#163)	0.29	0.062	0.012

4-4. NBO analysis of 2b (Table S4: based on the X-ray structure)

Intramolecular ChB was identified between the lone pair of carbonyl oxygen atom and the σ* orbital of S-C bond with interaction energies of 3.75 kcal/mol (entry 1). An additional intramolecular ChB ($LP_S \rightarrow \sigma^*_{S\text{–}N}$) was also observed in the equatorial form of **2b** (entry 2).

Table S4. Second order perturbation theory analysis of Fock matrix in NBO basis

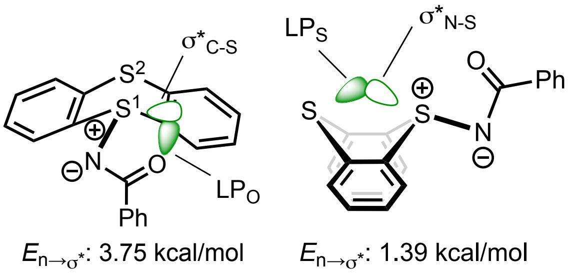

entry	Donor NBO of **2b** (i)	Acceptor NBO of **2b** (j)	E(2) kcal/mol	E(j) – E(i) a.u.	F(i,j) a.u.
1	LP O3<br>(Orbital#83)	BD* S2–C5<br>(Orbital#99)	3.75	0.55	0.041
2	LP S2<br>(Orbital#76)	BD*(1) S1–N<br>(Orbital#102)	1.39	0.62	0.027

5. Procedure for the protonation of 1 and 2a

5-Amino-5*H*-thianthren-5-ium trifluoromethanesulfonate (3)

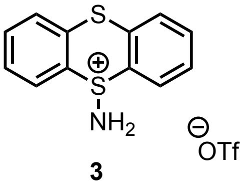

3

To a solution of 5-iminothianthrene (200 mg, 0.87 mmol) (**1**) in CH_2Cl_2 (8.7 mL), was added HOTf (100 μL, 1.13 mmol) dropwise at room temperature, and the reaction mixture was stirred at the same temperature for 21 h. After

volatiles are removed under reduced pressure, the residue was recrystallized from Et_2O to afford **3a** (212 mg, 64%) as white solid.

M.p. 117.8-118.9 ˚C (Et_2O); ^{1}H NMR (500 MHz, $CDCl_3$) δ 8.26 (m, 2H), 7.84 (m, 2H), 7.70 (m, 4H), 6.29 (brs, 2H); ^{13}C NMR (126 MHz, DMSO) δ 133.15, 131.50, 129.79, 129.45, 128.80, 128.53, 120.70 (q, J = 321.9 Hz); HRMS (EI): m/z calcd for $C_{12}H_{10}NS_2$ $[M]^+$ 232.0249, found 232.0234.

5-Amino-5*H*-thianthren-5-ium tetrafluoroborate (4)

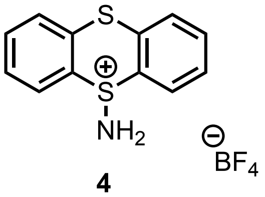

4

To a solution of 5-iminothianthrene (72 mg, 0.31 mmol) (**1**) in CH_2Cl_2 (3.0 mL), was added HBF_4 (40 µL, 0.62 mmol) dropwise at room temperature, and the reaction mixture was stirred at the same temperature for 1 h. After volatiles are removed under reduced pressure, the residue was recrystallized from Et_2O to afford **3b** (77 mg, 78%) as white solid.

M.p. 177.3-177.9 ˚C (Et_2O); ^{1}H NMR (500 MHz, $CDCl_3$) δ 8.30 (m, 2H), 7.86 (m, 2H), 7.73 (m, 4H), 5.58 (brs, 2H); ^{13}C NMR (75 MHz, DMSO) δ 133.61, 131.97, 130.23, 129.90, 129.27, 128.95; HRMS (EI): m/z calcd for $C_{12}H_{10}NS_2$ $[M]^+$ 232.0249, found 232.0243.

5-Acetamido-5*H*-thianthren-5-ium trifluoromethanesulfonate (5)

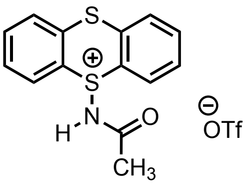

5

To a solution of **2a** (50 mg, 0.18 mmol) (**1**) in CH_2Cl_2 (5.0 mL), was added HOTf (20 µL, 0.20 mmol) dropwise at room temperature, and the reaction mixture was stirred at the same temperature for 24 h. After volatiles were removed under reduced pressure, the residue was recrystallized from Et_2O to afford **5** (212 mg, 85%) as white solid.

M.p. 129.0-130.2 ˚C (decomp.); ^{1}H NMR (500 MHz, $CDCl_3$) δ 11.62 (brs, 1H), 8.17 (d, J = 8.4 Hz, 2H), 7.90 (m, 2H), 7.81 (m, 2H), 7.67 (m, 2H), 2.18 (s, 3H); ^{13}C NMR (126 MHz, $CDCl_3$) δ 172.35, 137.82, 135.24, 133.14, 130.08, 129.23, 120.11 (q, J = 319.5 Hz), 117.88, 22.32; HRMS (EI): m/z calcd for $C_{14}H_{12}NOS_2$ $[M]^+$ 274.0355, found 274.0359.

Table S5. Summary of the crystal data of 3-5

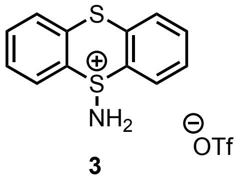

3
(ccdc: 2428009)

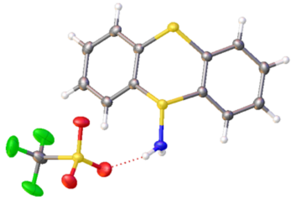

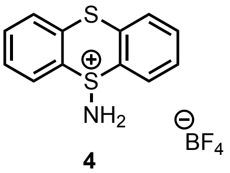

4
(ccdc: 2428010)

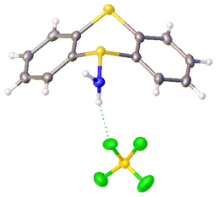

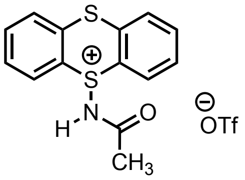

5
(ccdc: 2428012)

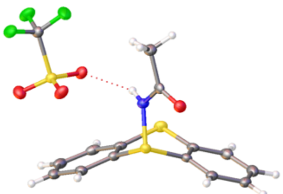

	3	4	5
CCDC	2428009	2428010	2428012
Empirical formula	$C_{13}H_{10}F_3NO_3S_3$	$C_{12}H_{10}BF_4NS_2$	$C_{15}H_{12}F_3NO_4S_3$
Formula weight	381.40	319.14	423.44
Temperature/K	100.00(10)	100.00(10)	100.00(10)
Crystal system	Triclinic	Monoclinic	Monoclinic
Space group	P-1	$P2_1/c$	$P2_1/n$
a/Å	6.7740(2)	6.9823(2)	8.7420(2)
b/Å	10.6202(4)	11.5538(3)	9.3586(3)
c/Å	12.0129(4)	16.2894(4)	20.6584(4)
α/°	116.071(3)	90	90
β/°	94.093(3)	90.410(2)	93.145(2)
γ/°	91.827(3)	90	90
Volume/Å^3	772.38(5)	1314.07(6)	1687.58(7)
Z	2	4	4
ρ_{calc}g/cm^3	1.640	1.613	1.667
μ/mm^{-1}	4.833	4.028	4.539
F(000)	388.0	648.0	864.0
Crystal size/mm^3	0.07 × 0.07 × 0.01	0.1 × 0.1 × 0.03	0.2 × 0.02 × 0.01
Radiation	Cu Kα (λ = 01.54184)	Cu Kα (λ = 1.54184)	Cu Kα (λ = 1.54184)
2Θ range for data collection/°	8.232 to 156.002	9.384 to 154.322	8.574 to 155.038
Index (h, k, lmax)	6, 13, 14	8, 11, 19	8, 10, 25
Reflections collected	7876	12825	11750
Independent reflections	3126 [R_{int} = 0.0203, R_{sigma} = 0.0261]	2678 [R_{int} = 0.0382, R_{sigma} = 0.0266]	3419 [R_{int} = 0.0364, R_{sigma} = 0.0334]
Goodness-of-fit on F^2	1.083	1.152	1.056
Final R indexes [I>=2σ (I)]	R_1 = 0.0324, wR_2 = 0.0840	R_1 = 0.0734, wR_2 = 0.0266	R_1 = 0.0355, wR_2 = 0.0876
Final R indexes [all data]	R_1 = 0.0345, wR_2 = 0.0853	R_1 = 0.0779, wR_2 = 0.1853	R_1 = 0.0400, wR_2 = 0.0901

6. Reaction of 1 with iso(thio)cyanate

6-1. General procedure of the reaction of 1 with iso(thio)cyanate

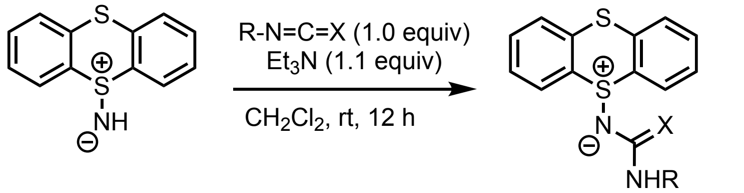

1

6 (X = O), **7** (X = S)

To a solution of **1** (1.0 equiv) and Et_3N (1.1 equiv) in CH_2Cl_2 (0.1 M), was added iso(thio)cyanate (1.0 equiv) dropwise at room temperature, and the reaction mixture was stirred for 12-14 h. After volatiles were removed under reduced pressure, the residue was recrystallized from hexane to afford the corresponding adducts.

1-(5λ^4-Thianthren-5-ylidene)-3-phenylurea (6a)

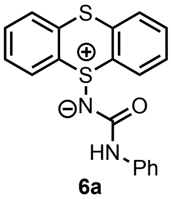

6a

Yield: 70%; M.p. 180.1-194.2 ˚C (CH_2Cl_2/*n*-hexane); ^{1}H NMR (500 MHz, $CDCl_3$) δ 7.97 (dd, *J* = 7.9, 1.4 Hz, 2H), 7.69 (dd, *J* = 7.6, 1.2 Hz, 2H), 7.58 – 7.52 (m, 4H), 7.49 (td, *J* = 7.5, 1.4 Hz, 2H), 7.34 – 7.29 (m, 2H), 7.01 (tt, *J* = 7.4, 1.2 Hz, 1H); ^{13}C NMR (75 MHz, $CDCl_3$) δ 164.42, 140.29, 134.30, 131.39, 130.79, 129.52, 129.06, 128.81, 126.53, 122.32, 118.53; HRMS (EI): *m*/*z* calcd for $C_{19}H_{14}N_2OS_2$ $[M]^+$ 350.0548, found 350.0541.

<u>NOTE</u>

The gradual degradation of compound **7** was observed during the acquisition of the ^{13}C NMR spectrum. Additionally, mass spectrometric analysis exhibited $[M–248]^+$ signals, indicating that compound **7** undergoes degradation with the loss of thianthrene (216) and a sulfur atom (32). Therefore, only ^{1}H NMR and IR spectra were reported in the supporting information.

1-(5λ^4-Thianthren-5-ylidene)-3-phenylthiourea (7a)

Yield: 80%; ^{1}H NMR (500 MHz, $CDCl_3$) δ 8.22 (brs, 1H), 8.00 (d, *J* = 6.9 Hz, 2H), 7.74 – 7.71 (m, 2H), 7.64 (d, *J* = 7.8 Hz, 2H), 7.54 – 7.49 (m, 4H), 7.33 (t, *J* = 7.9 Hz, 2H), 7.10 (t, *J* = 7.4 Hz, 1H); IR (neat) : 3214, 3177, 3112, 3020, 1593, 1525, 1495, 1436, 1373, 1312, 1299, 1231, 1180, 1131, 1113, 1080, 1026, 1010, 954, 901, 865, 840, 750, 725, 708, 691, 679, 638, 621, 599, 580, 541, 514, 499,451, 424, 412 cm^{-1}.

1-(5λ^4-Thianthren-5-ylidene)-3-(4-methoxyphenyl)thiourea (7b)

Yield: 75%; ^{1}H NMR (500 MHz, $CDCl_3$) δ 8.12 (brs, 1H), 7.95 (m, 2H), 7.70 (d, *J* = 7.7 Hz, 2H), 7.51 (m, 6H), 6.89 (d, *J* = 8.6 Hz, 2H), 3.81 (s, 3H); IR (neat): 3196, 3174, 3105, 3023, 2990, 2943, 2825, 1532, 1504, 1435, 1421, 1380, 1318, 1297, 1267, 1242, 1228, 1183, 1169, 1125, 1112, 1083, 1035, 1010, 961, 920, 865, 850, 818, 758, 749, 722,

711, 700, 678, 645, 633, 582, 550, 515, 497, 471, 445, 426, 418, 409 cm^{-1}.

1-(5λ^4-Thianthren-5-ylidene)-3-(4-nitrophenyl)thiourea (7c)

Yield: 88%; ^{1}H NMR (500 MHz, $CDCl_3$) δ 8.26 (brs, 1H), 8.15 (d, *J* = 9.0 Hz, 2H), 8.10 – 8.05 (m, 2H), 7.80 – 7.76 (m, 2H), 7.70 (d, *J* = 9.0 Hz, 2H), 7.60 – 7.53 (m, 4H); IR (neat) : 3371, 3107, 3065, 2351, 1592, 1529, 1498, 1481, 1425, 1372, 1362, 1317, 1300, 1241, 1168, 1159, 1121, 1108, 1024, 1010, 968, 946, 865, 846, 828, 814, 755, 745, 730, 710, 686, 671, 654, 635, 624, 587, 542, 527, 512, 493, 456, 438, 409 cm^{-1}.

1-(5λ^4-Thianthren-5-ylidene)-3-(2-bromophenyl)thiourea (7d)

Yield: 72%; ^{1}H NMR (500 MHz, $CDCl_3$) δ 8.46 (brs, 1H), 8.41 (d, *J* = 8.2 Hz, 1H), 7.99 (dd, *J* = 7.1, 2.0 Hz, 2H), 7.72 (dd, *J* = 7.0, 1.9 Hz, 2H), 7.58 (dd, *J* = 7.9, 1.5 Hz, 1H), 7.55 – 7.47 (m, 4H), 7.29 (d, *J* = 8.2 Hz, 1H), 6.97 (t, *J* = 7.4 Hz, 1H); IR (neat) : 3136, 3066, 2987, 2953, 1568, 1507, 1464, 1434, 1372, 1275, 1249, 1227, 1189, 1155, 1128, 1113, 1026, 973, 944, 933, 865, 842, 755, 744, 732, 694, 658, 640, 601, 579, 541, 518, 507, 495, 477, 459, 450, 441, 422 cm^{-1}.

1-(5λ^4-Thianthren-5-ylidene)-3-(3,5-bis(trifluoromethyl)phenyl)thiourea (7e)

Yield: 64%; ^{1}H NMR (500 MHz, $CDCl_3$) δ 8.16 (brs, 1H), 8.09 – 8.03 (m, 4H), 7.79 – 7.74 (m, 2H), 7.55 (ddd, *J* = 6.7, 3.9, 1.8 Hz, 4H), 7.49 (s, 1H); IR (neat) : 3220, 3068, 2994, 1620, 1550, 1469, 1455, 1435, 1376, 1325, 1275, 1231, 1161, 1121, 1039, 998, 956, 936, 891, 881, 843, 747, 733, 715, 700, 682, 656, 636, 607, 574, 541, 526, 518, 501, 485, 460, 425, 414, 405 cm^{-1}.

1-(5λ^4-Thianthren-5-ylidene)-3-(2-pyridyl)thiourea (7f)

Yield: 83%; ^{1}H NMR (500 MHz, $CDCl_3$) δ 8.80 (s, 1H), 8.43 (s, 1H), 8.30 (d, *J* = 4.9 Hz, 1H), 8.05 (dd, *J* = 7.2, 2.1 Hz, 2H), 7.74 (dd, *J* = 7.0, 2.1 Hz, 2H), 7.63 (t, *J* = 7.8 Hz, 1H), 7.53 (ddd, *J* = 6.3, 3.6, 1.8 Hz, 4H), 7.00 – 6.93 (m, 1H); IR (neat) : 3157, 3064, 2971, 1597, 1571, 1513, 1470, 1431, 1308, 1262, 1223, 1154, 1114, 1087, 1054, 1014, 997, 971, 940, 870, 858, 806, 780, 757, 731, 678, 618, 595, 540, 512, 497, 454, 410 cm^{-1}.

6-2. X-ray structure of 6a and 7a

Table S6. Summary of the crystal data of 6a and 7a

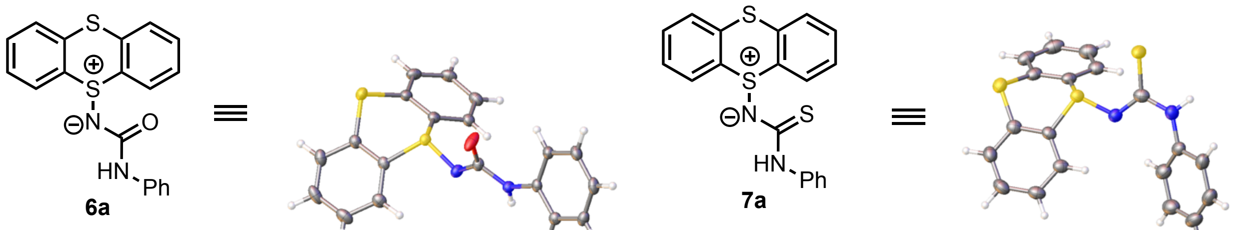

(ccdc:2428013) (ccdc: 2428014)

	6a	7a
CCDC	2428013	2428014
Empirical formula	$C_{19}H_{14}N_2OS_2$	$C_{19}H_{14}N_2S_3$
Formula weight	350.44	366.50
Temperature/K	100.00(10)	100.00(10)
Crystal system	Orthorhombic	Monoclinic
Space group	$P2_12_12_1$	$P2_1/c$
a/Å	5.04550(10)	11.2221(12)
b/Å	11.1663(2)	11.6933(13)
c/Å	28.8009(6)	13.8415(13)
α/°	90	90
β/°	90	108.261(10)
γ/°	90	90
Volume/Å^3	1622.63	1724.9(3)
Z	4	4
ρ_{calc}g/cm^3	1.435	1.411
μ/mm^{-1}	3.034	3.936
F(000)	728.0	760.0
Crystal size/mm^3	0.1 × 0.05 × 0.03	0.05 × 0.05 × 0.01
Radiation	Cu Kα (λ = 1.54184)	Cu Kα (λ = 1.54184)
2Θ range for data collection/°	6.138 to 155.32	8.296 to 158.414
Index (h, k, lmax)	5, 13, 35	14, 13, 16
Reflections collected	6160	11147
Independent reflections	3019 [R_{int} = 0.0287, R_{sigma} = 0.0365]	3515 [R_{int} = 0.0954, R_{sigma} = 0.0819]
Goodness-of-fit on F^2	1.033	1.046
Final R indexes [I>=2σ (I)]	R_1 = 0.0303, wR_2 = 0.0773	R_1 = 0.0789, wR_2 = 0.2192
Final R indexes [all data]	R_1 = 0.0327, wR_2 = 0.0785	R_1 = 0.0996, wR_2 = 0.2345

6-3. NBO analysis of 6a (Table S7)

Intramolecular ChB was identified between the lone pair of carbonyl oxygen atom and the σ* orbital of S-C bond with interaction energies of 0.76 kcal/mol (entry 1). An additional intramolecular ChB ($LP_S \rightarrow \sigma^*_{S\text{–}N}$) was also observed in the equatorial form of **6a** (entry 2).

Table S7. Second order perturbation theory analysis of Fock matrix in NBO basis

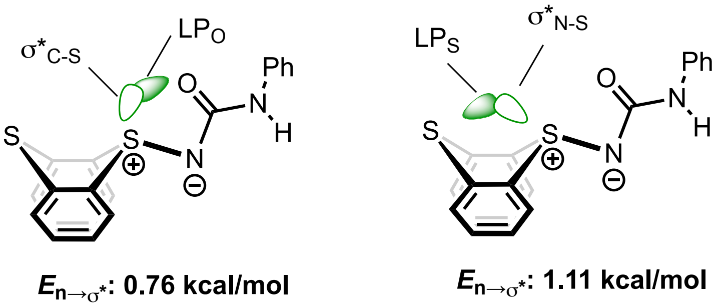

entry	Donor NBO of **6a** (i)	Acceptor NBO of **6a** (j)	E(2) kcal/mol	E(j) – E(i) a.u.	F(i,j) a.u.
1	LP O3<br>(Orbital#86)	BD* S1–C15<br>(Orbital#103)	0.76	0.53	0.018
2	LP S2<br>(Orbital#80)	BD* S1–N5<br>(Orbital#106)	1.11	0.65	0.025

6-4. NBO analysis of 7a (Table S8)

Intramolecular ChB was identified between the lone pair of thiocarbonyl sulfur atom and the σ* orbital of S-C bond with interaction energies of 5.35 kcal/mol (entry 1). An additional intramolecular ChB ($LP_S \rightarrow \sigma^*_{S\text{–}N}$) was also observed in the equatorial form of **7a** (entry 2).

Table S8. Second order perturbation theory analysis of Fock matrix in NBO basis

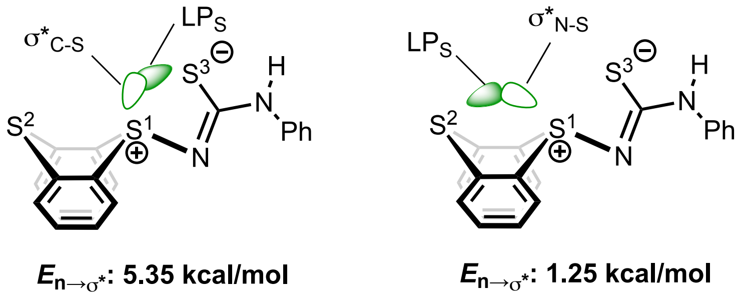

entry	Donor NBO of **7a** (i)	Acceptor NBO of **7a** (j)	E(2) kcal/mol	E(j) – E(i) a.u.	F(i,j) a.u.
1	LP S3<br>(Orbital#94)	BD* S1–C7<br>(Orbital#107)	5.35	0.48	0.046
2	LP S2<br>(Orbital#84)	BD* S1–N4<br>(Orbital#110)	1.25	0.62	0.026

7. General procedure for the synthesis of cyanamide from 7

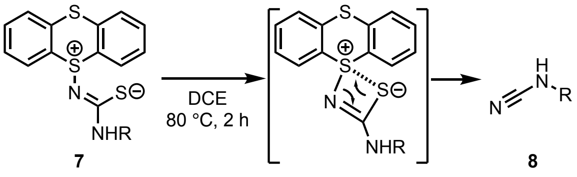

A solution of **7** (0.10 mmol) in DCE (1.0 mL) was heated at 80 ˚C for 2 hours. The resulting mixture was directly purified by silica gel column chromatography (*n*-hexane/AcOEt = 1/1) to give **8**.

N-Phenylcyanamide (8a)[42]

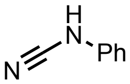

8a

Yield: 79%; ^{1}H NMR (500 MHz, $CDCl_3$) δ 7.40 – 7.32 (m, 2H), 7.14 – 7.07 (m, 1H), 7.06 – 6.98 (m, 2H), 5.96 (brs, 1H). The spectral data were corresponded to the literature data.[42]

N-(4-Methoxyphenyl)cyanamide (8b)[42]

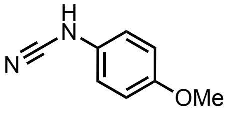

8b

Yield: 65%; ^{1}H NMR (500 MHz, $CDCl_3$) δ 6.95 (d, J = 9.0 Hz, 2H), 6.88 (d, J = 9.0 Hz, 2H), 5.52 (brs, 1H), 3.79 (s, 3H). The spectral data were corresponded to the literature data.[42]

N-(4-Nitrophenyl)cyanamide (8c)[43]

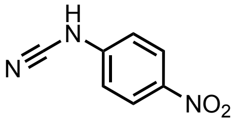

8c

Yield: 93%; ^{1}H NMR (500 MHz, DMSO) δ 11.16 (s, 1H), 8.21 (d, J = 8.7 Hz, 2H), 7.10 (d, J = 8.6 Hz, 2H). The spectral data were corresponded to the literature data.[43]

N-(2-Bromophenyl)cyanamide (8d)[42]

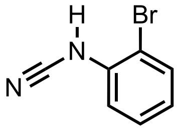

8d

Yield: 89%; ^{1}H NMR (500 MHz, $CDCl_3$) δ 7.52 (dd, J = 8.1, 1.4 Hz, 1H), 7.37 (td, J = 7.8, 1.4 Hz, 1H), 7.31 (dd, J = 8.1, 1.6 Hz, 1H), 6.99 (td, J = 7.7, 1.5 Hz, 1H), 6.32 (brs, 1H). The spectral data were corresponded to the literature data.[42]

***N*-(3,5-Bis(trifluoromethyl)phenyl)cyanamide (8e)**

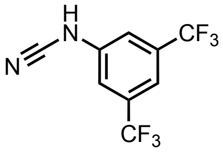

8e

Yield: 75%; Mp 180.1-194.2℃ (EtOAc/*n*-hexane). ^{1}H NMR (500 MHz, $CDCl_3$) δ 7.62 (s, 1H), 7.46 (d, J = 2.9 Hz, 2H), 5.30 (brs, 1H); ^{13}C NMR (75 MHz, $CDCl_3$) δ 139.08, 133.78 (q, J = 34.1 Hz), 122.80 (q, J = 272.9 Hz), 118.03–117.45 (m), 115.59, 109.05; HRMS (EI): *m/z* calcd for $C_9H_4F_6N_2$ $[M]^+$ 254.0279, found 254.0286.

8. Reaction of 2 as *N*-acylnitrene equivalents

8.1 Reaction of 2c with phenylacetylene under photo-irradiation

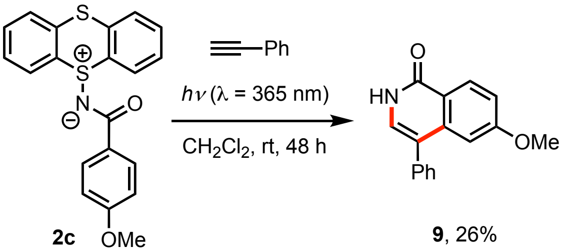

2c

9, 26%
(ccdc: 2428016)

A solution of **2c** (30 mg, 0.082 mmol) and phenylacetylene (90 μL, 0.82 mmol) in CH_2Cl_2 (3.0 mL) was photo-irradiated ($h\nu$ = 365 nm) for 48 hours. After the solvent was removed under reduced pressure, the residue was purified by preparative thin layer chromatography (*n*-hexane/AcOEt = 1/1) to give **9** (5.4 mg, 26%).

6-Methoxy-4-phenylisoquinolin-1(2*H*)-one (9)[34]

^{1}H NMR (500 MHz, $CDCl_3$) δ 8.42 (d, J = 8.8 Hz, 1H), 7.51 – 7.45 (m, 2H), 7.42 (t, J = 6.8 Hz, 3H), 7.10 (dd, J = 9.1, 2.5 Hz, 1H), 7.00 (s, 1H), 6.94 (d, J = 2.5 Hz, 1H), 3.79 (s, 3H).

8.2. Reaction of 2b with triphenylphosphine under photo-irradiation (Scheme S1)

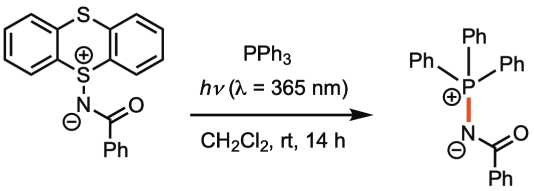

2b

10, 16%

A solution of **2b** (28.6 mg, 0.0853 mmol) and triphenylphosphine (70.6 mg, 0.27 mmol) in CH_2Cl_2 (5.0 mL) was photo-irradiated ($h\nu$ = 365 nm) for 14 hours. After the solvent was removed under reduced pressure, the residue was purified by preparative thin layer chromatography (*n*-hexane/AcOEt = 2/3) to give **10** (5.3 mg, 16%).

N-(Triphenyl-λ^5-phosphaneylidene)benzamide (10)[44]

^{1}H NMR (500 MHz, $CDCl_3$) δ 8.34 (d, J = 7.4 Hz, 2H), 7.89 – 7.80 (m, 6H), 7.57 (t, J = 7.7 Hz, 3H), 7.48 (t, J = 8.0 Hz, 6H), 7.44 (d, J = 7.1 Hz, 1H), 7.40 (t, J = 7.7 Hz, 2H). The spectral data were consistent with the literature data.[44]

Table S9. Summary of the crystal data of 8d and 9

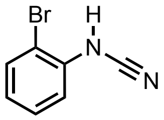

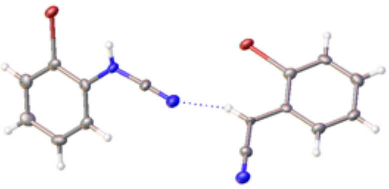

8d (ccdc: 2428015)

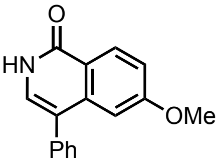

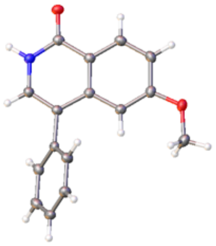

9

(ccdc: 2428016)

	**8d**	**9**
CCDC	2428015	2428016
Empirical formula	$C_{15}H_{10}Br_2N_3$	$C_{16}H_{13}NO_2$
Formula weight	392.08	251.27
Temperature/K	100.00(10)	100.00(10)
Crystal system	Monoclinic	Monoclinic
Space group	Pc	$P2_1/c$
a/Å	12.3737(4)	6.56956(10)
b/Å	3.92980(10)	24.4809(4)
c/Å	14.6108(4)	7.90465(13)
α/°	90	90
β/°	95.464(3)	102.5116(16)
γ/°	90	90
Volume/Å^3	707.24(4)	1241.11(3)
Z	2	4
ρ_{calc}g/cm^3	1.841	1.345
μ/mm^{-1}	7.204	0.718
F(000)	382.0	528.0
Crystal size/mm^3	0.15 × 0.02 × 0.02	0.2 × 0.1 × 0.1
Radiation	Cu Kα (λ = 1.54184)	Cu Kα (λ = 1.54184)
2Θ range for data collection/°	7.176 to 154.348	7.222 to 155.214
Index (h, k, lmax)	14, 1, 17	8, 30, 9
Reflections collected	3363	16678
Independent reflections	2027 [R_{int} = 0.0405, R_{sigma} = 0.0457]	2556 [R_{int} = 0.0180, R_{sigma} = 0.0098]
Goodness-of-fit on F^2	1.075	1.082
Final R indexes [I>=2σ (I)]	R_1 = 0.0432, wR_2 = 0.1155	R_1 = 0.0341, wR_2 = 0.0928
Final R indexes [all data]	R_1 = 0.0438, wR_2 = 0.1162	R_1 = 0.0359, wR_2 = 0.0942

9. References

[35] T. Fujita, T. Maeda, B. J. Kim, A. Tatami, D. Miyamoto, H. Kawaguchi, N. Tsuchiya, M. Yoshida, W. Kawashima, H. Morita, "Photolytic aziridination by thianthrene sulfilimine derivatives" *J. Sulfur Chem.* **2008**, *29*, 459-465.

[36] O. V. Dolomanov, L. J. Bourhis, R. J. Gildea, J. A. K. Howard, H. Puschmann, "OLEX2: a complete structure solution, refinement and analysis program" *J. Appl. Cryst.* **2009**, *42*, 339-341.

[37] G.M. Sheldrick, "SHELXT – Integrated space-group and crystal-structure determination" *Acta Cryst.* **2015**, *A71*, 3-8.

[38] G. M. Sheldrick, "Crystal structure refinement with SHELXL" *Acta Cryst.* **2015**, *C71*, 3-8.

[39] J.-D. Chai, M. Head-Gordon, "Long-range corrected hybrid density functionals with damped atom-atom dispersion corrections," *Phys. Chem. Chem. Phys.*, **2008**, *10*, 6615-6620.

[40] Gaussian 16, Revision C.01, M. J. Frisch, G. W. Trucks, H. B. Schlegel, G. E. Scuseria, M. A. Robb, J. R. Cheeseman, G. Scalmani, V. Barone, G. A. Petersson, H. Nakatsuji, X. Li, M. Caricato, A. V. Marenich, J. Bloino, B. G. Janesko, R. Gomperts, B. Mennucci, H. P. Hratchian, J. V. Ortiz, A. F. Izmaylov, J. L. Sonnenberg, D. Williams-Young, F. Ding, F. Lipparini, F. Egidi, J. Goings, B. Peng, A. Petrone, T. Henderson, D. Ranasinghe, V. G. Zakrzewski, J. Gao, N. Rega, G. Zheng, W. Liang, M. Hada, M. Ehara, K. Toyota, R. Fukuda, J. Hasegawa, M. Ishida, T. Nakajima, Y. Honda, O. Kitao, H. Nakai, T. Vreven, K. Throssell, J. A. Montgomery, Jr., J. E. Peralta, F. Ogliaro, M. J. Bearpark, J. J. Heyd, E. N. Brothers, K. N. Kudin, V. N. Staroverov, T. A. Keith, R. Kobayashi, J. Normand, K. Raghavachari, A. P. Rendell, J. C. Burant, S. S. Iyengar, J. Tomasi, M. Cossi, J. M. Millam, M. Klene, C. Adamo, R. Cammi, J. W. Ochterski, R. L. Martin, K. Morokuma, O. Farkas, J. B. Foresman, and D. J. Fox, Gaussian, Inc., Wallingford CT, 2016.

[41] G. Henkelman and H. Jónsson, "A climbing image nudged elastic band method for finding saddle points and minimum energy paths", *J. Chem. Phys.* **2000**, *113*, 9901-9904.

[42] G. Zhang, Y. Zhao, C. Ding, "A cascade process for directly converting nitriles (RCN) to cyanamides (RNHCN) via SO_2F_2-activated Tiemann rearrangement" *Org. Biomol. Chem.*, **2019**, *17*, 7684-7688.

[43] K. Škoch, I. Císařová, P. Štěpnička, "Selective gold-catalysed synthesis of cyanamides and 1-substituted 1*H*-tetrazol-5-amines from isocyanides" *Chem. Eur. J.* **2018**, *24*, 13788 – 13791.

[44] Q. Zhang, S. Liu, Y. Xie, T. Du, S. Wang, J. Guan, J. Li, H. Tang, Z. Zhou, "Examining 1,4,2-dioxazol-5-one as an alternative reagent to acyl azides in the Staudinger reaction" *Eur. J. Org. Chem.* **2024**, *27*, e202400930.

10. Copies of ^{1}H and ^{13}C NMR charts

_N_-(5λ⁴-Thianthren-5-ylidene)acetamide (2a)[27]

^{1}H-NMR (500 MHz, $CDCl_3$)

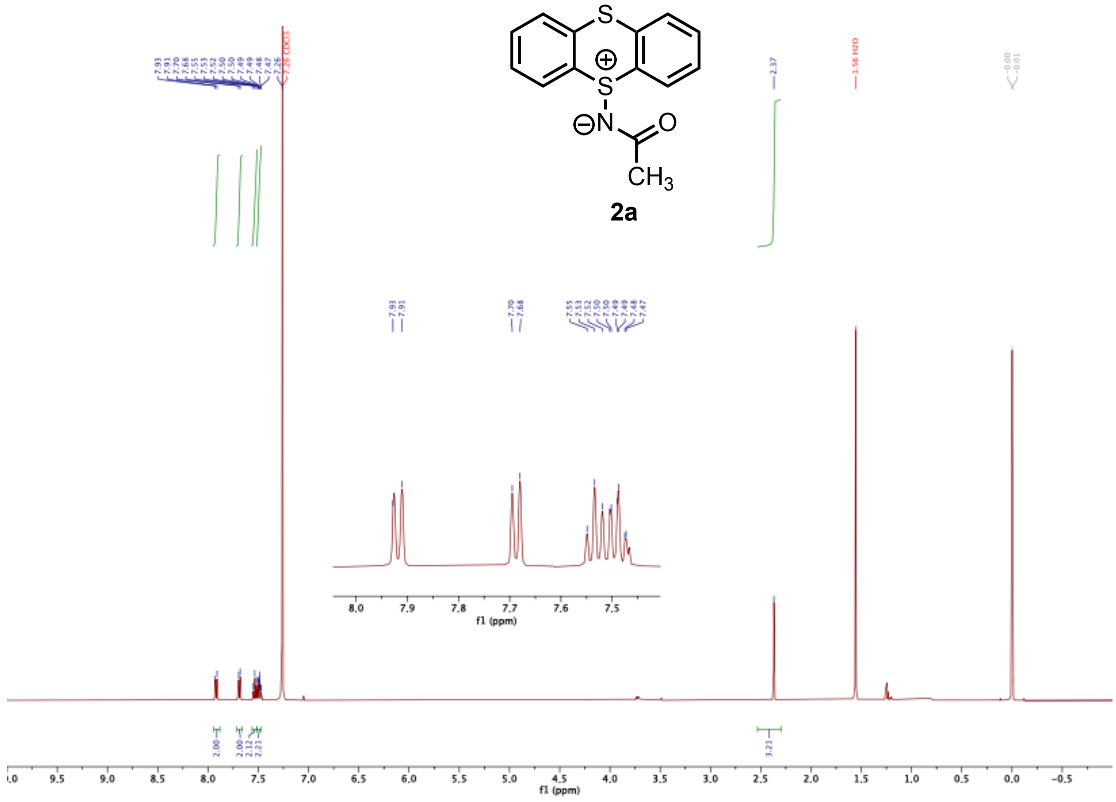

_N_-(5λ⁴-Thianthren-5-ylidene)benzamide (2b) [27]

^{1}H-NMR (500 MHz, $CDCl_3$)

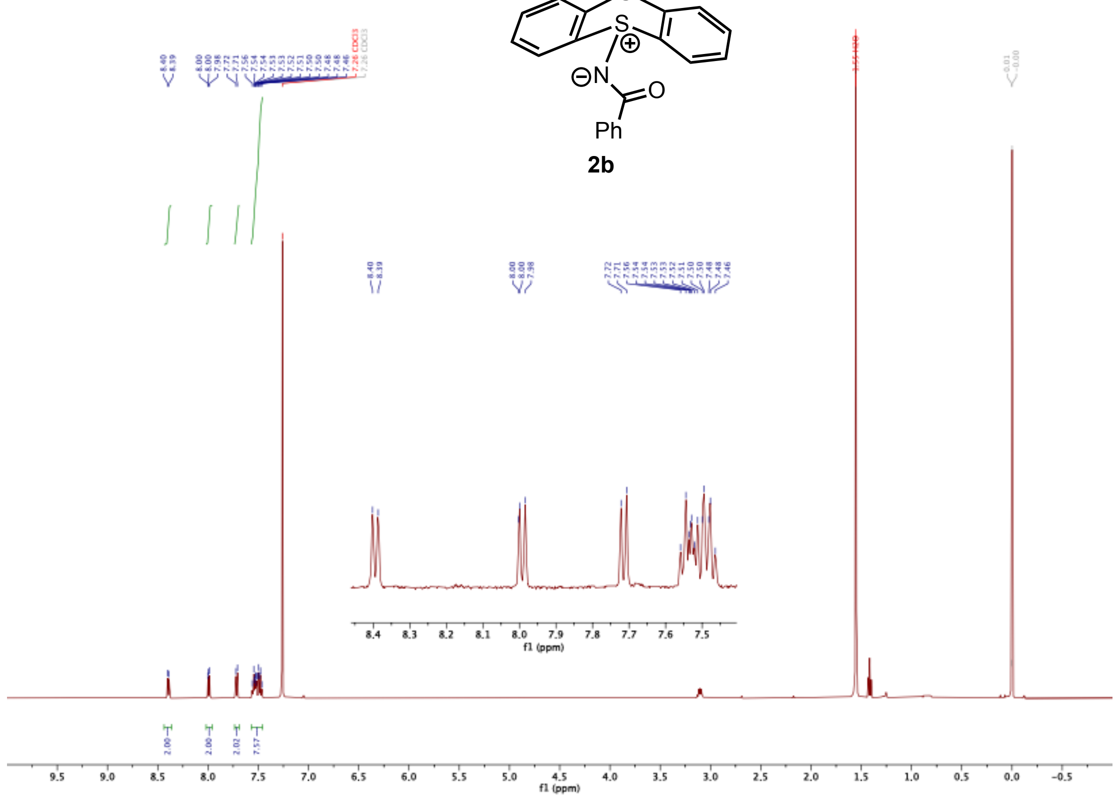

N-(5λ^4-Thianthren-5-ylidene)benzamide (2c) [35]

^{1}H-NMR (500 MHz, $CDCl_3$)

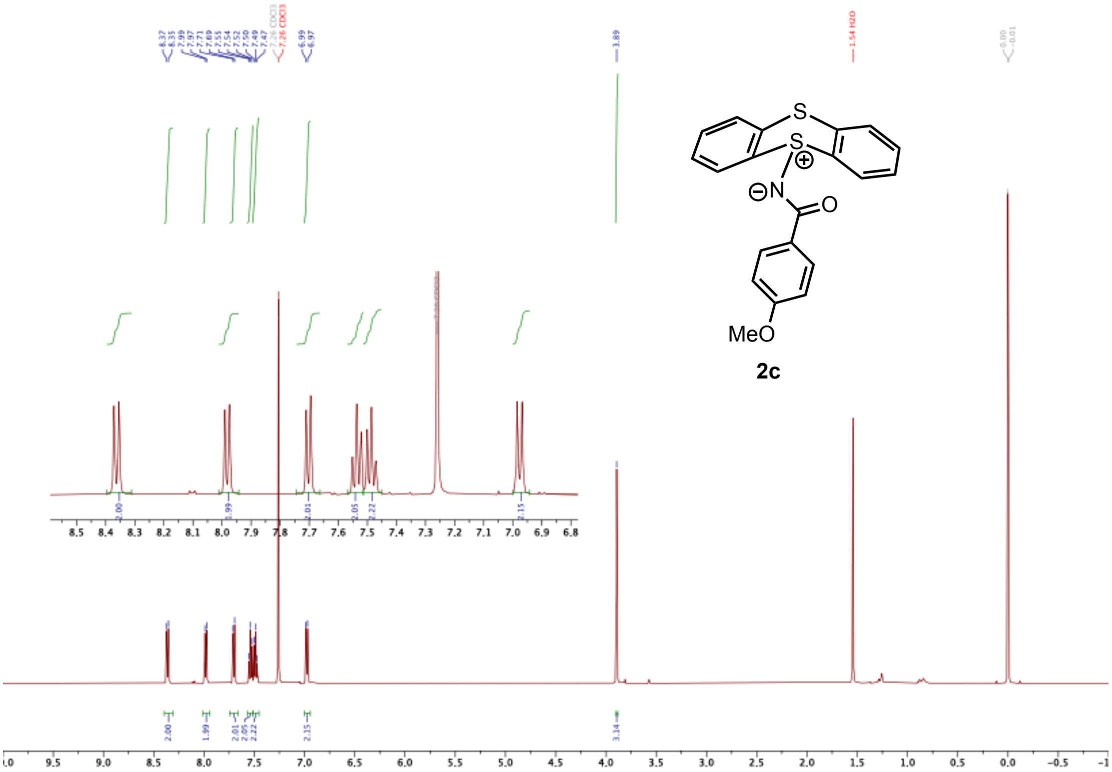

N-(5λ^4-Thianthren-5-ylidene)-2,2,2-trifluoroacetamide (2d)

^{1}H-NMR (500 MHz, $CDCl_3$)

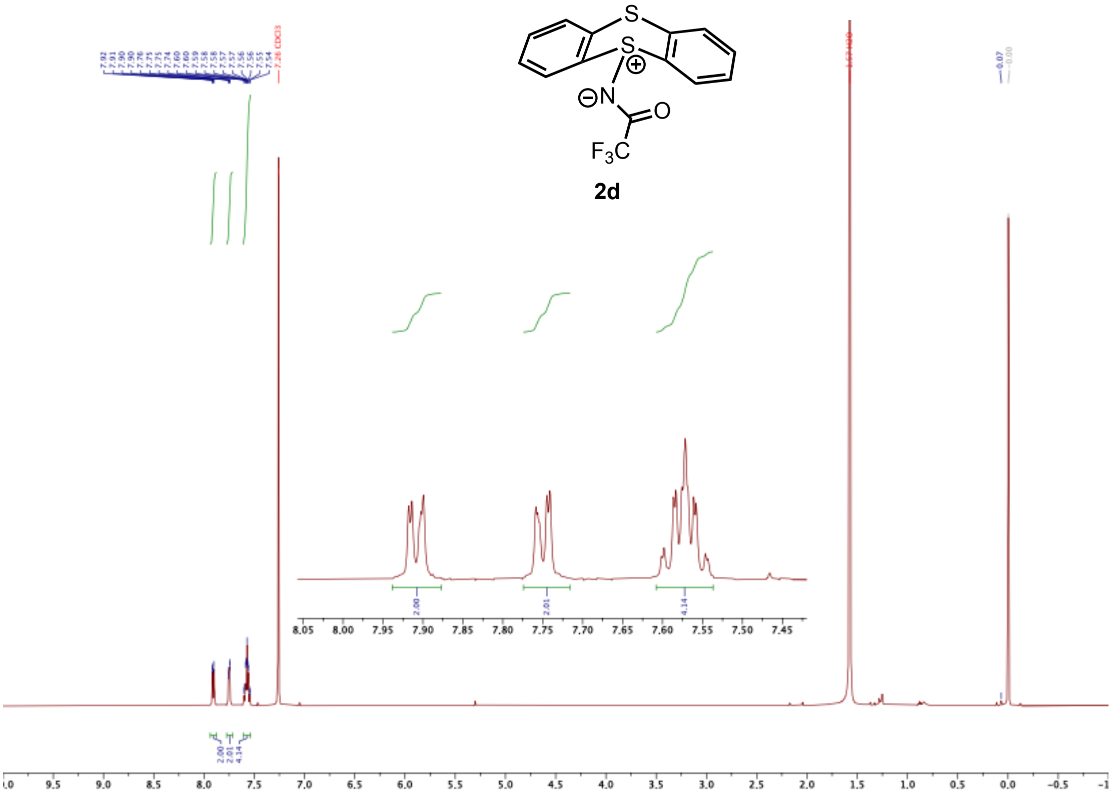

N-(5λ^4-Thianthren-5-ylidene)-2,2,2-trifluoroacetamide (2d)

^{13}C-NMR (75 MHz, $CDCl_3$)

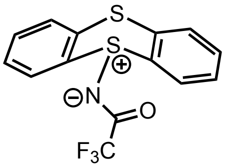

2d

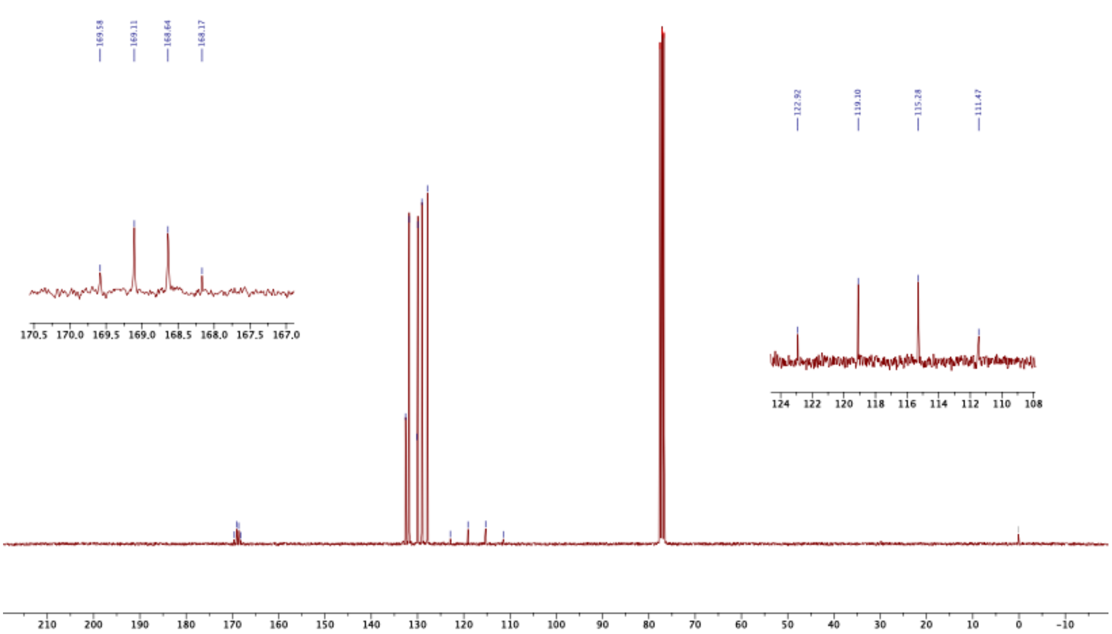

N-(5λ^4-Thianthren-5-ylidene)-2,2,2-trifluoroacetamide (2d)

^{19}F-NMR (282 MHz, $CDCl_3$)

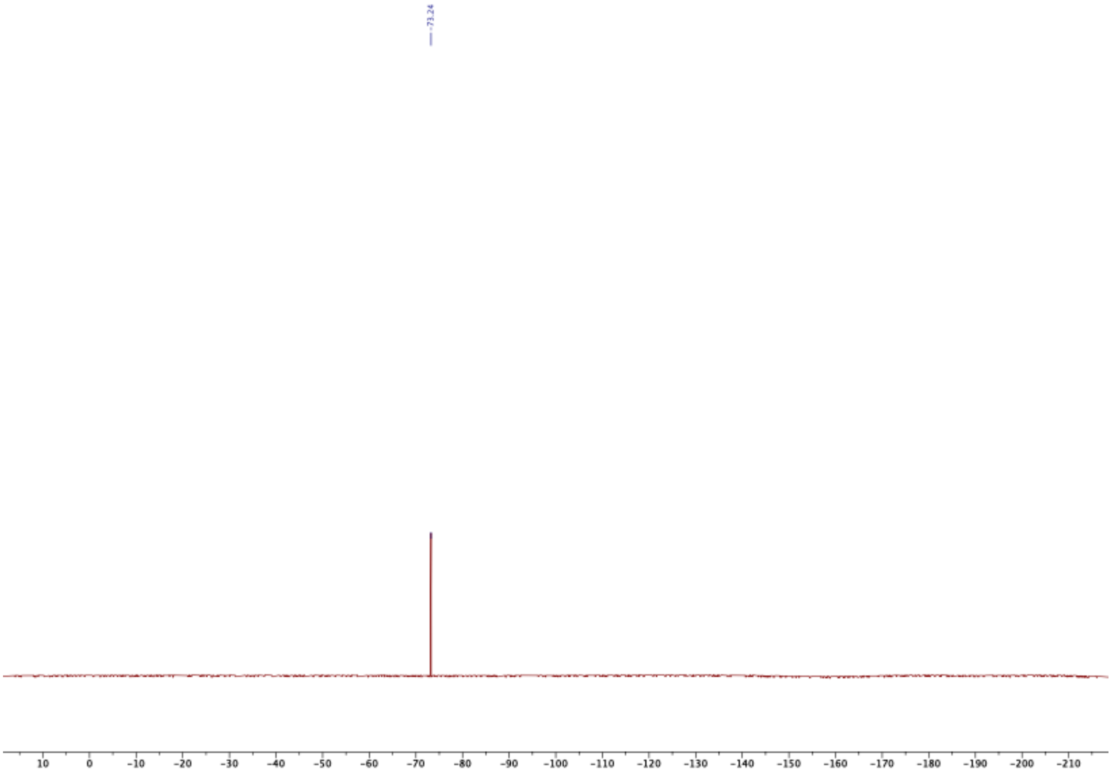

***tert*-Butyl (5λ^4-thianthren-5-ylidene)carbamate (2e)**

^{1}H-NMR (500 MHz, $CDCl_3$)

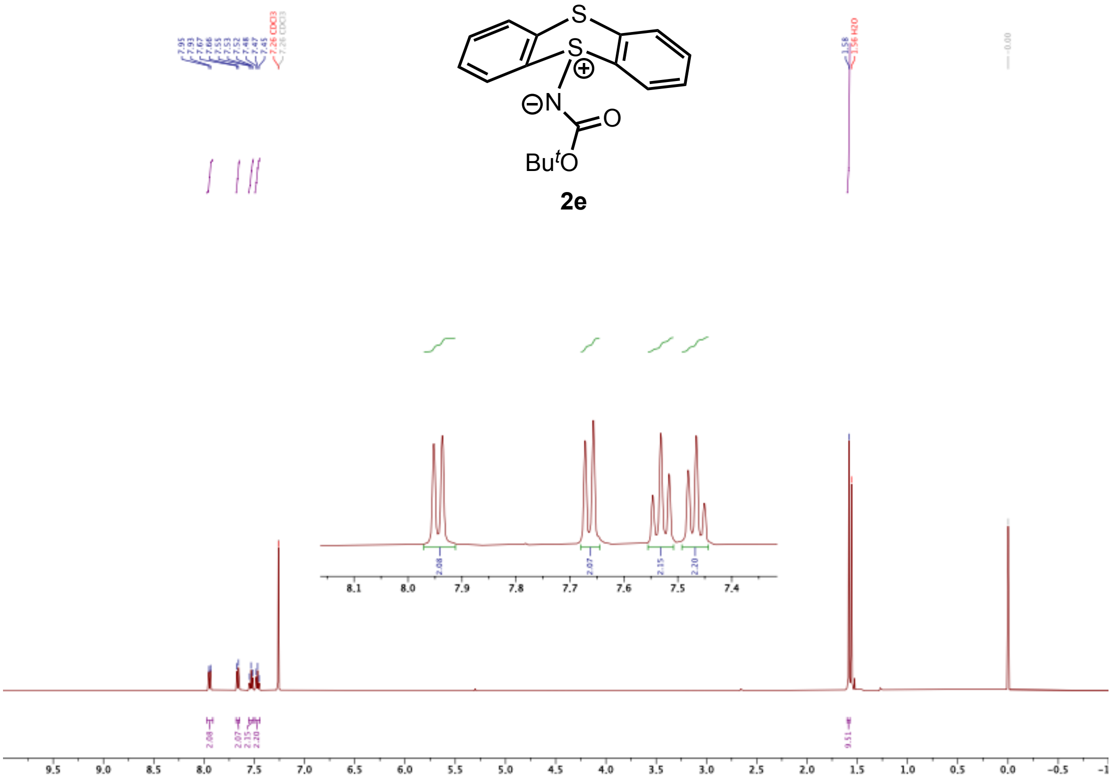

***tert*-Butyl (5λ^4-thianthren-5-ylidene)carbamate (2e)**

^{13}C-NMR (75 MHz, $CDCl_3$)

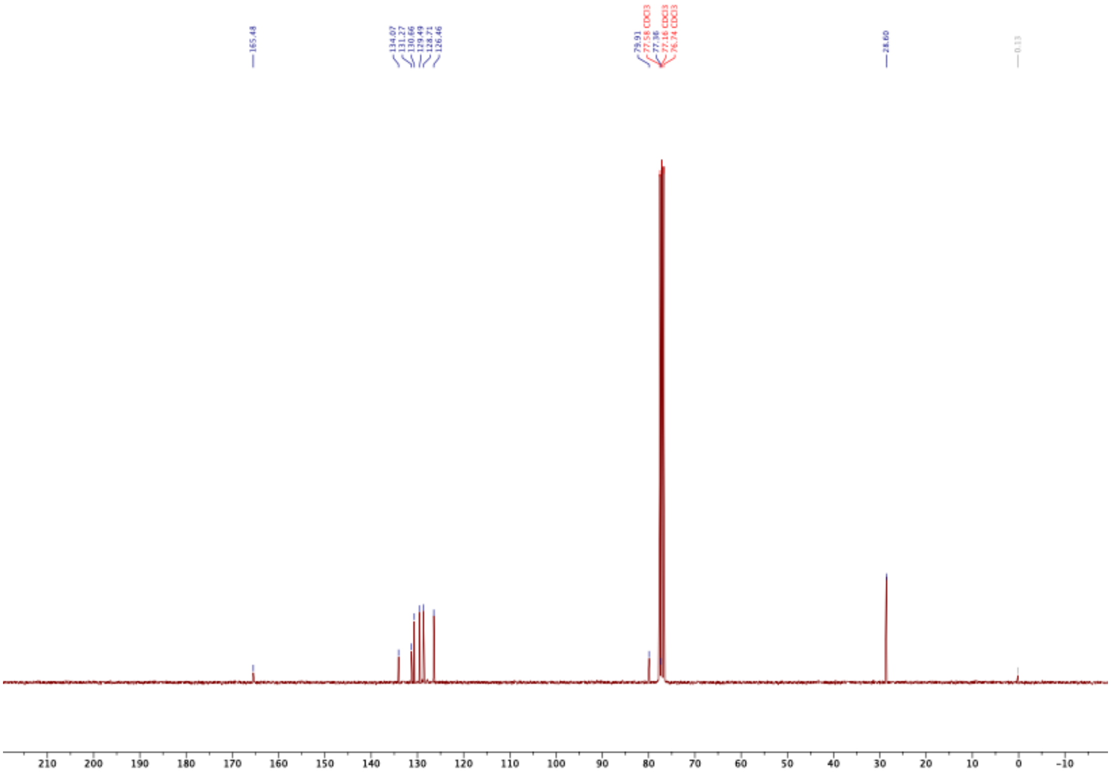

5-Amino-5*H*-thianthren-5-ium trifluoromethanesulfonate (3)

^{1}H NMR (500 MHz, $CDCl_3$)

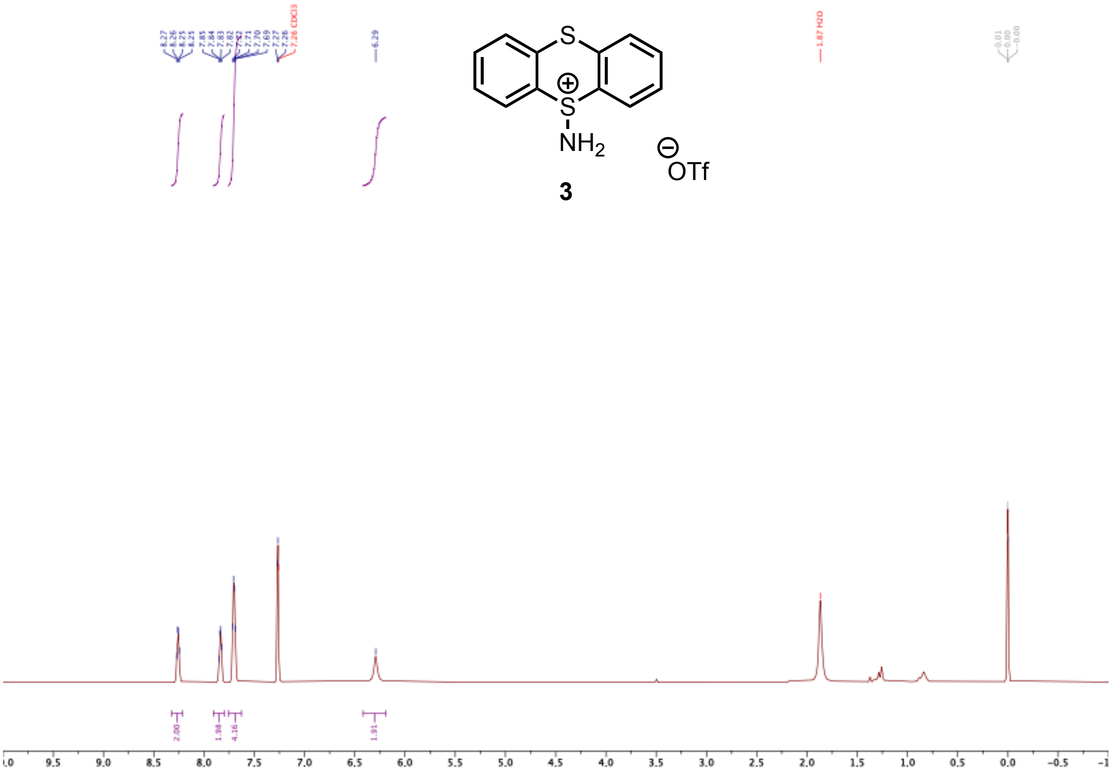

5-Amino-5*H*-thianthren-5-ium trifluoromethanesulfonate (3)

^{13}C NMR (126 MHz, DMSO)

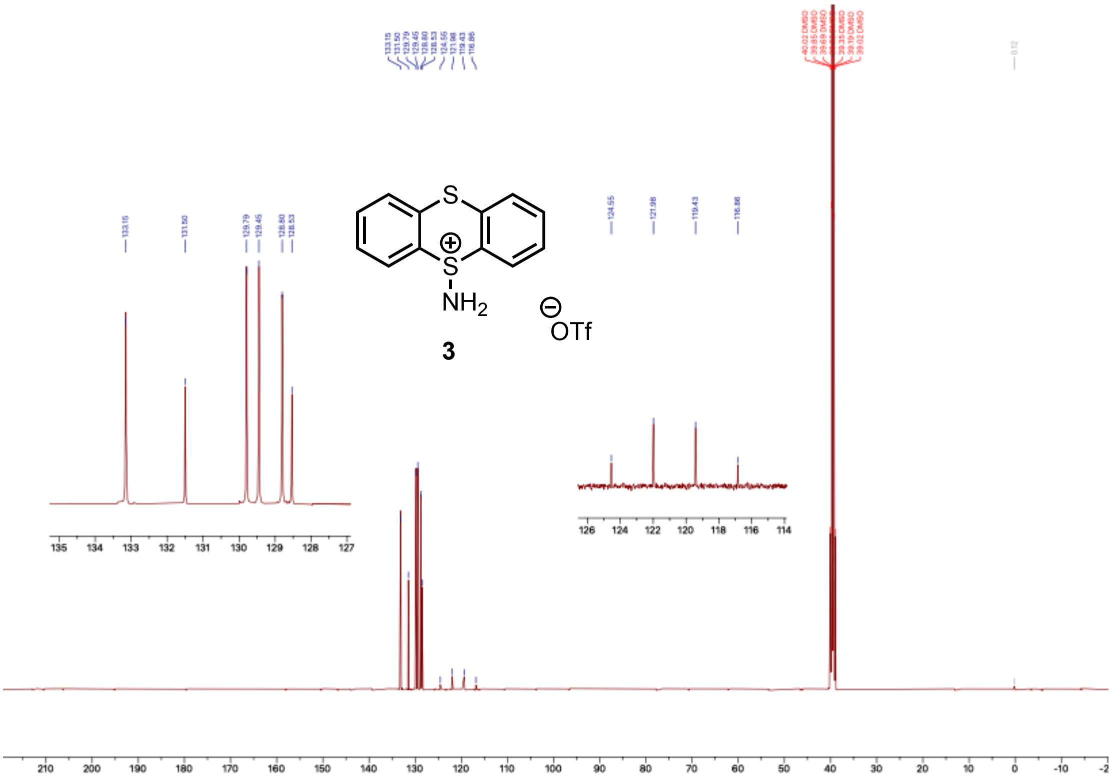

5-Amino-5*H*-thianthren-5-ium tetrafluoroborate (4)

^{1}H NMR (500 MHz, $CDCl_3$)

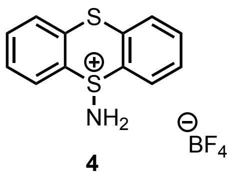

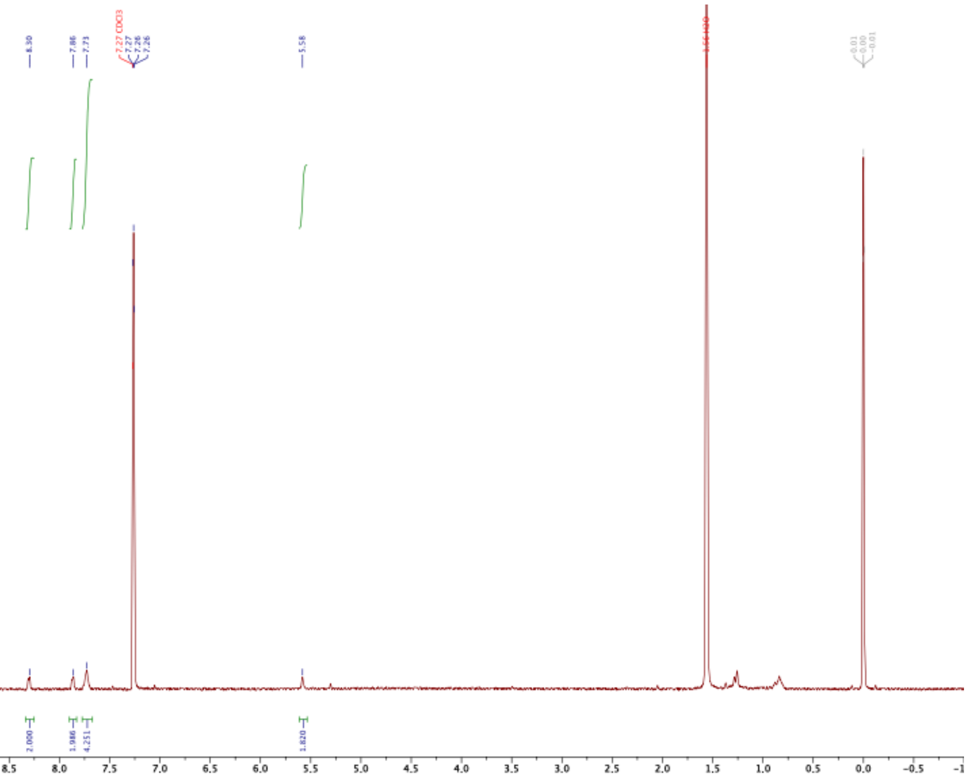

5-Amino-5*H*-thianthren-5-ium tetrafluoroborate (4)

^{13}C NMR (75 MHz, DMSO)

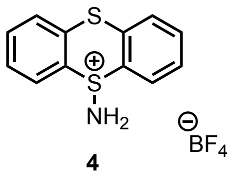

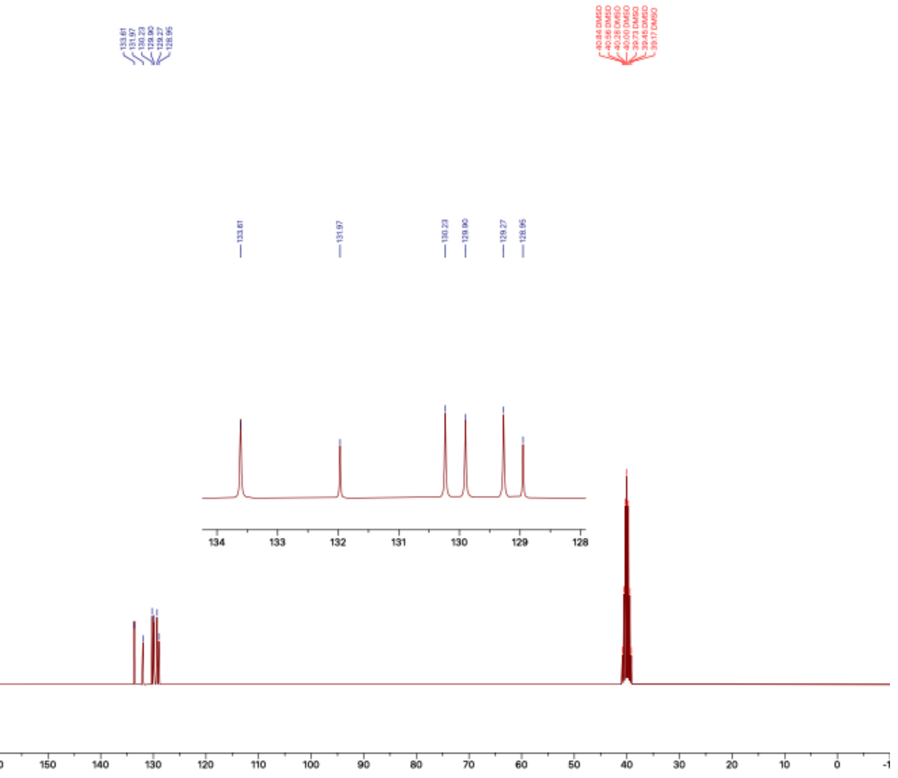

5-Acetamido-5*H*-thianthren-5-ium trifluoromethanesulfonate (5)

^{1}H NMR (500 MHz, $CDCl_3$)

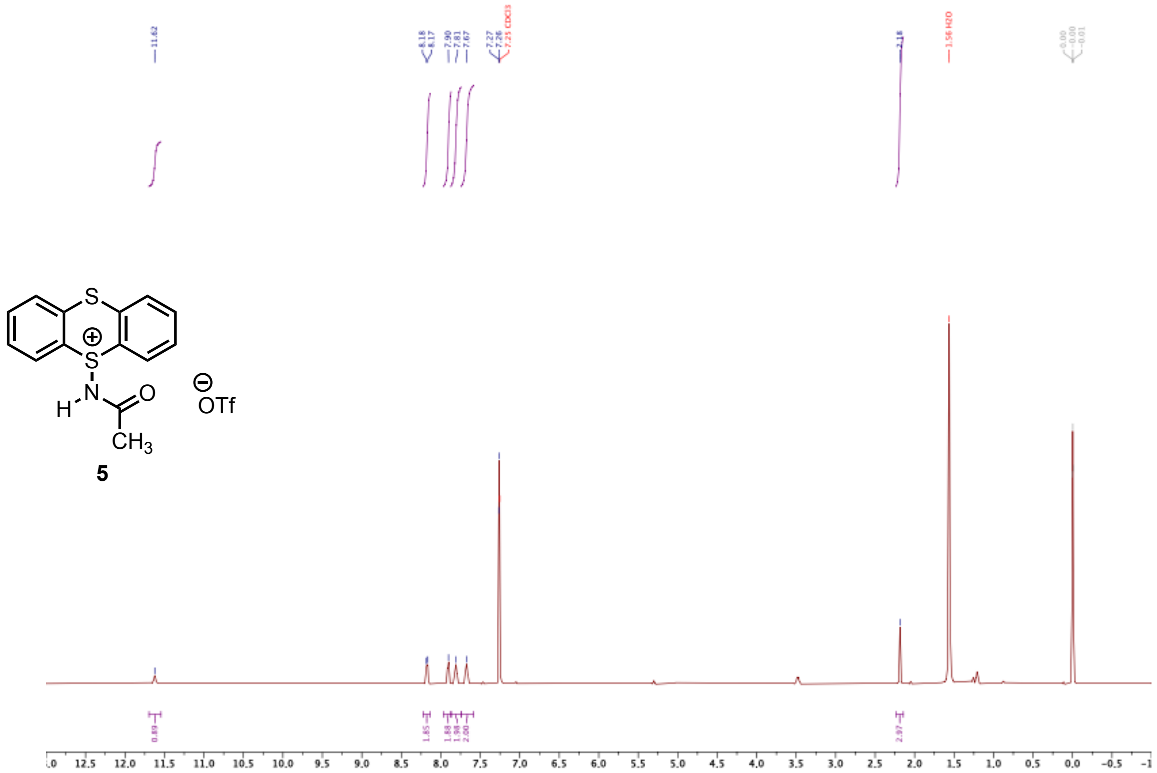

5-Acetamido-5*H*-thianthren-5-ium trifluoromethanesulfonate (5)

^{13}C NMR (126 MHz, $CDCl_3$)

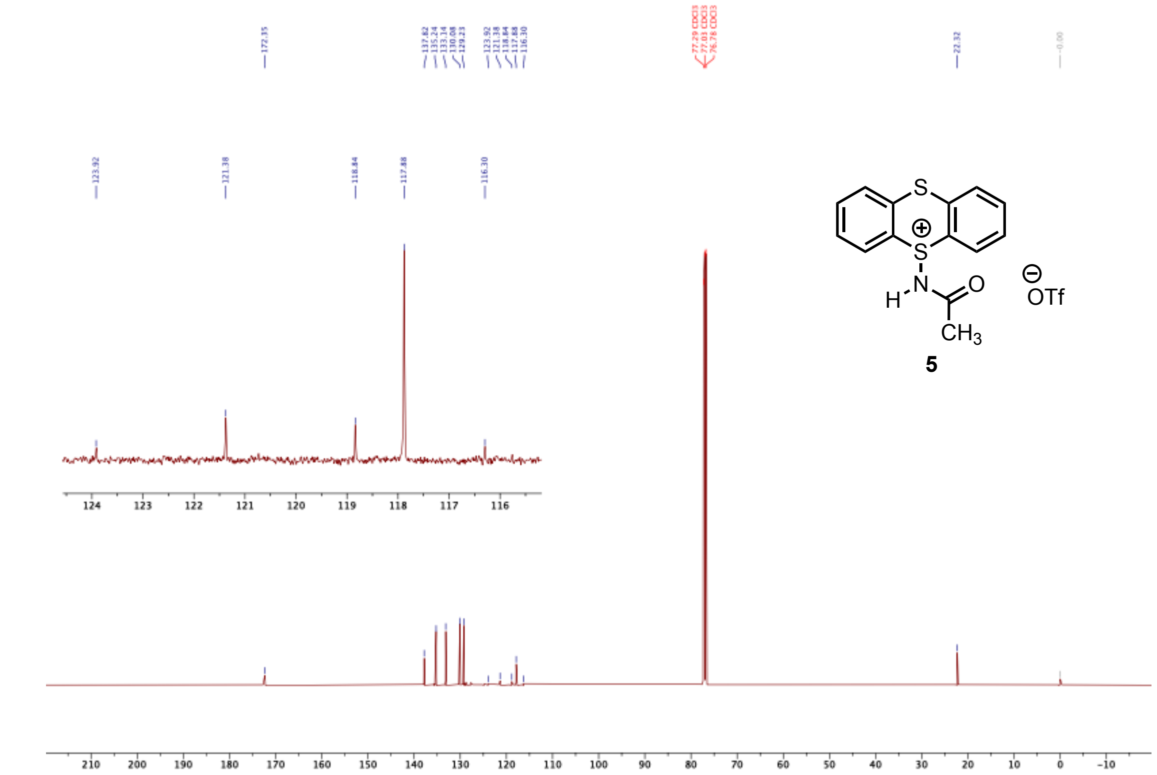

1-(5λ⁴-Thianthren-5-ylidene)-3-phenylurea (6a)

^{1}H-NMR (500 MHz, $CDCl_3$)

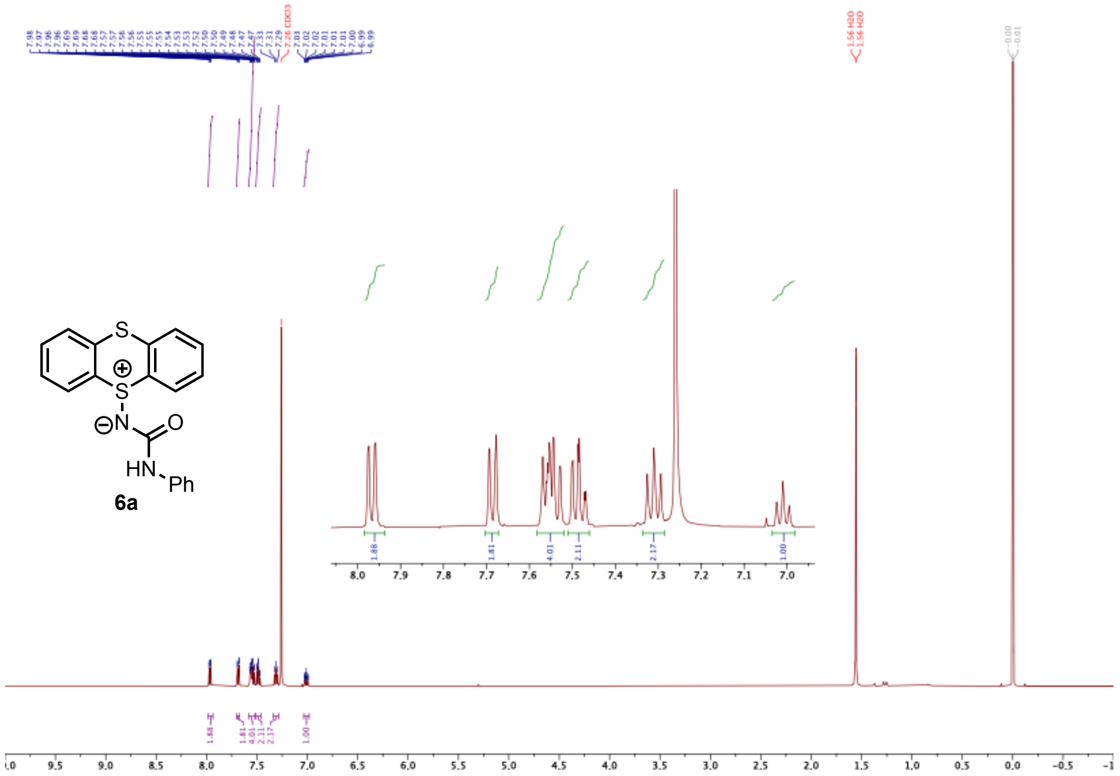

1-(5λ⁴-Thianthren-5-ylidene)-3-phenylurea (6a)

^{13}C-NMR (75 MHz, $CDCl_3$)

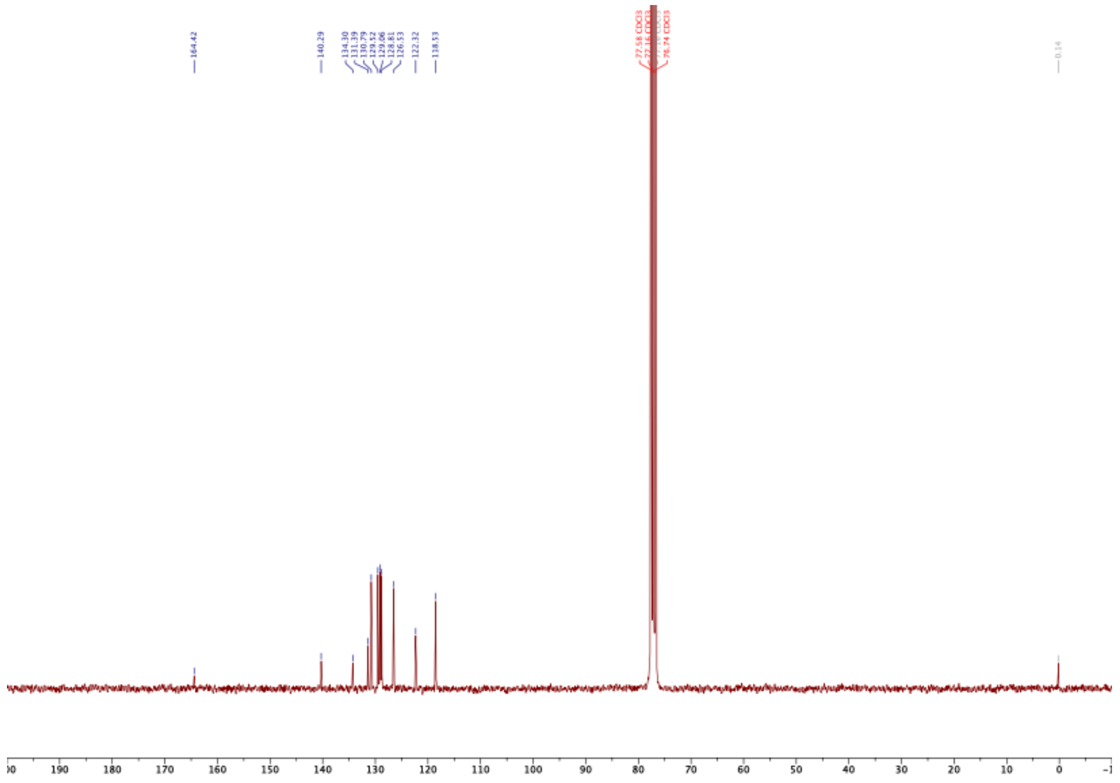

1-(5λ⁴-Thianthren-5-ylidene)-3-phenylthiourea (7a)

^{1}H NMR (500 MHz, CDCl$_3$)

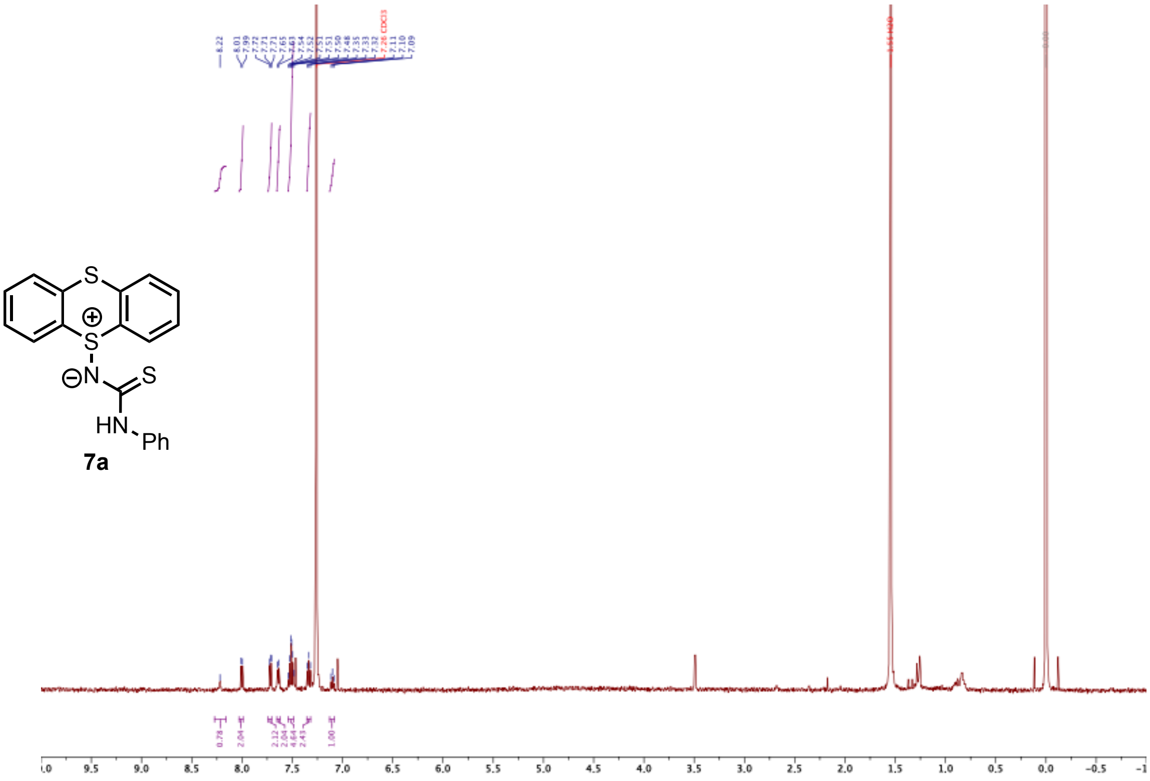

1-(5λ⁴-Thianthren-5-ylidene)-3-(4-methoxyphenyl)thiourea (7b)

^{1}H NMR (500 MHz, CDCl$_3$)

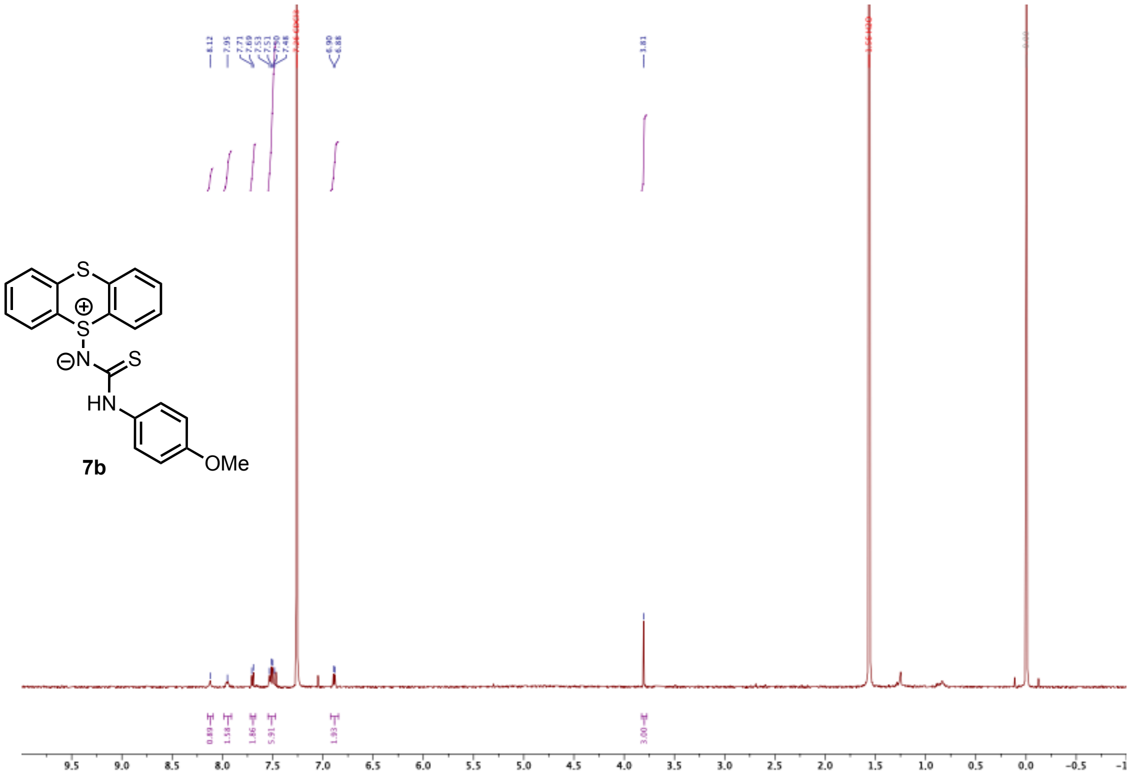

1-(5λ^4-Thianthren-5-ylidene)-3-(4-nitrophenyl)thiourea (7c)

^{1}H NMR (500 MHz, $CDCl_3$)

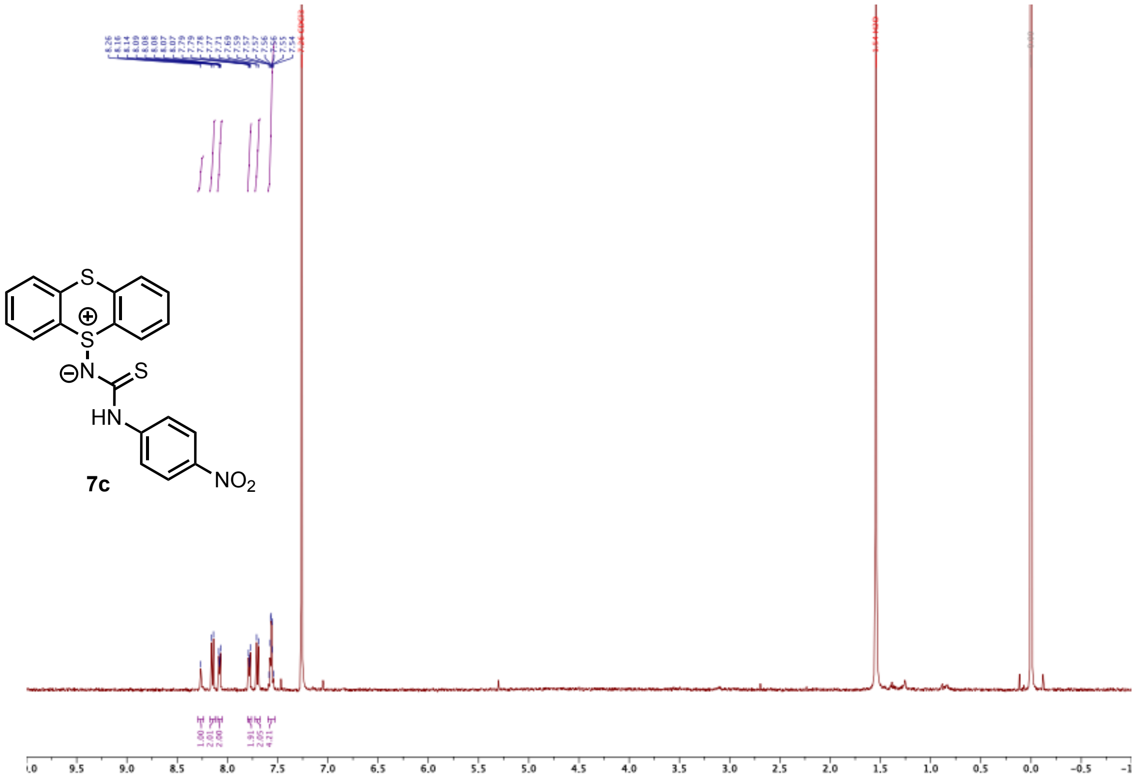

1-(5λ^4-Thianthren-5-ylidene)-3-(2-bromophenyl)thiourea (7d)

^{1}H NMR (500 MHz, $CDCl_3$)

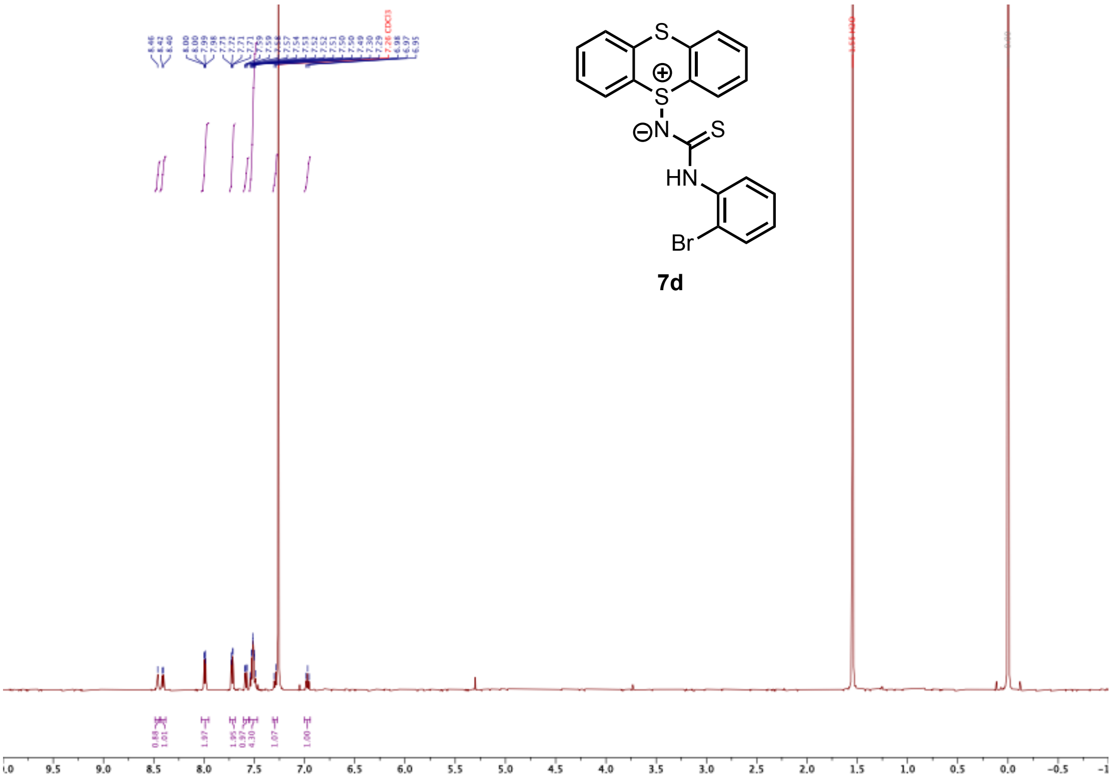

1-(5λ⁴-Thianthren-5-ylidene)-3-(3,5-bis(trifluoromethyl)phenyl)thiourea (7e)

^{1}H NMR (500 MHz, $CDCl_3$)

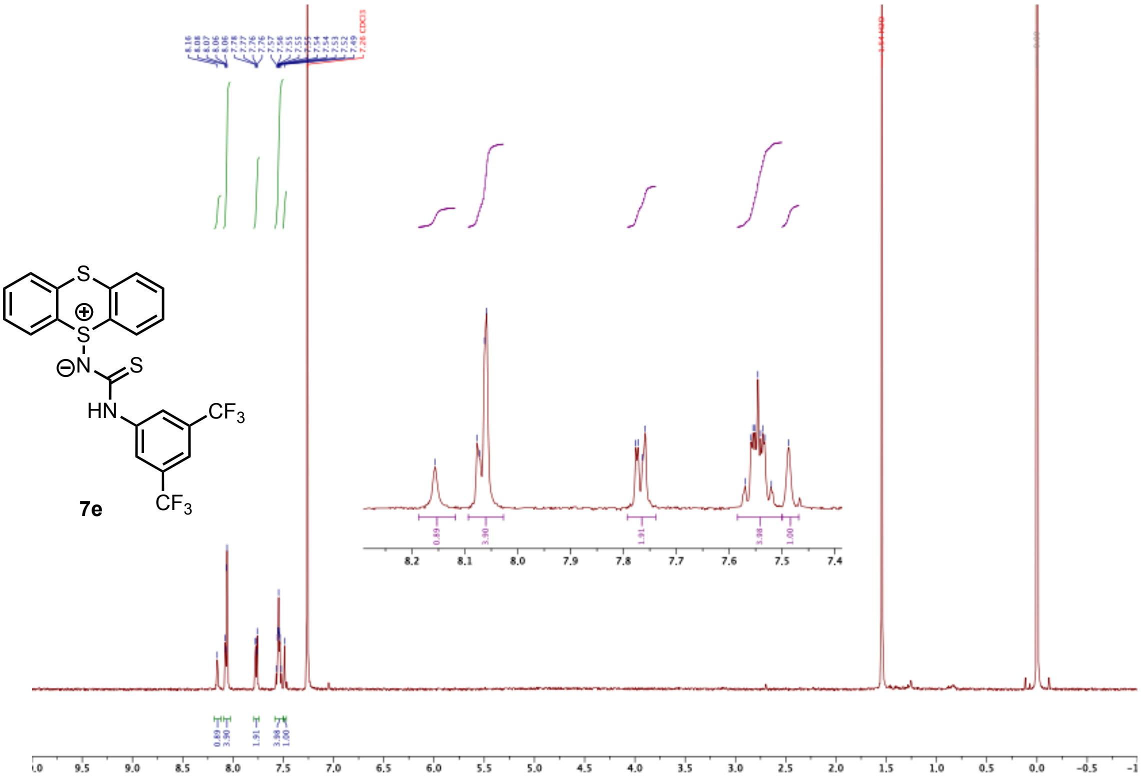

1-(5λ⁴-Thianthren-5-ylidene)-3-(2-pyridyl)thiourea (7f)

^{1}H NMR (500 MHz, $CDCl_3$)

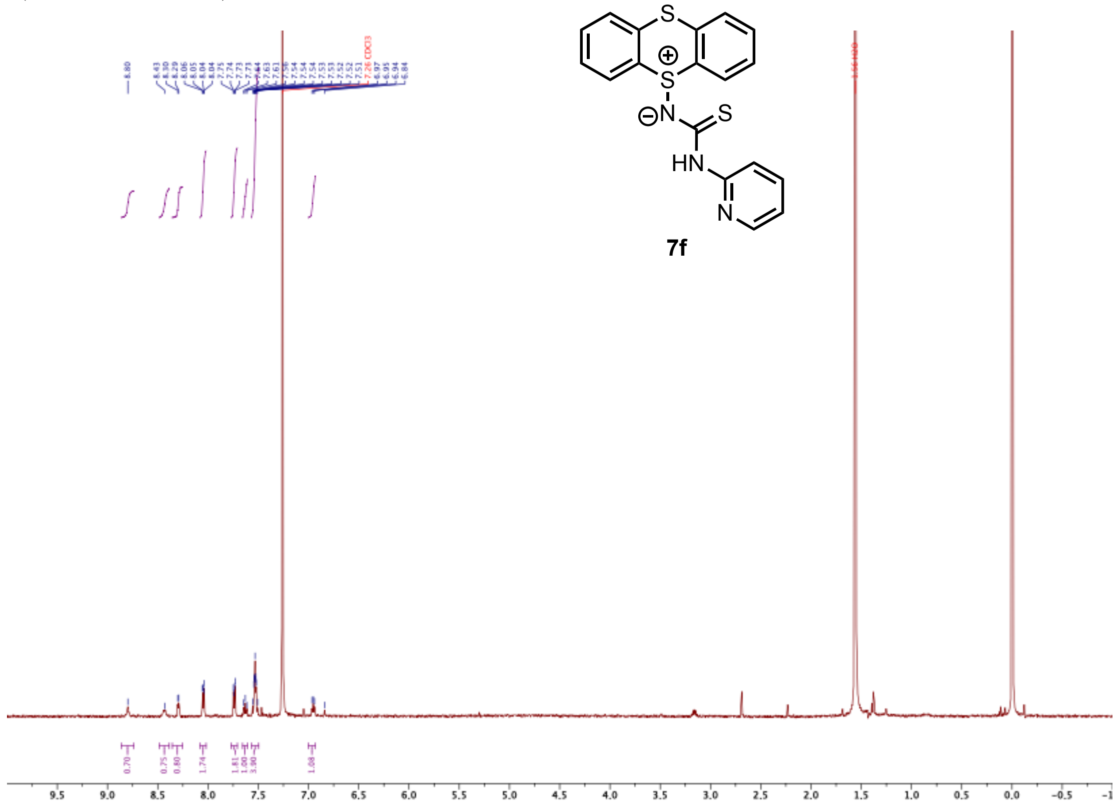

***N*-Phenylcyanamide (8a)** [42]

^{1}H NMR (500 MHz, $CDCl_3$)

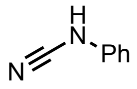

8a

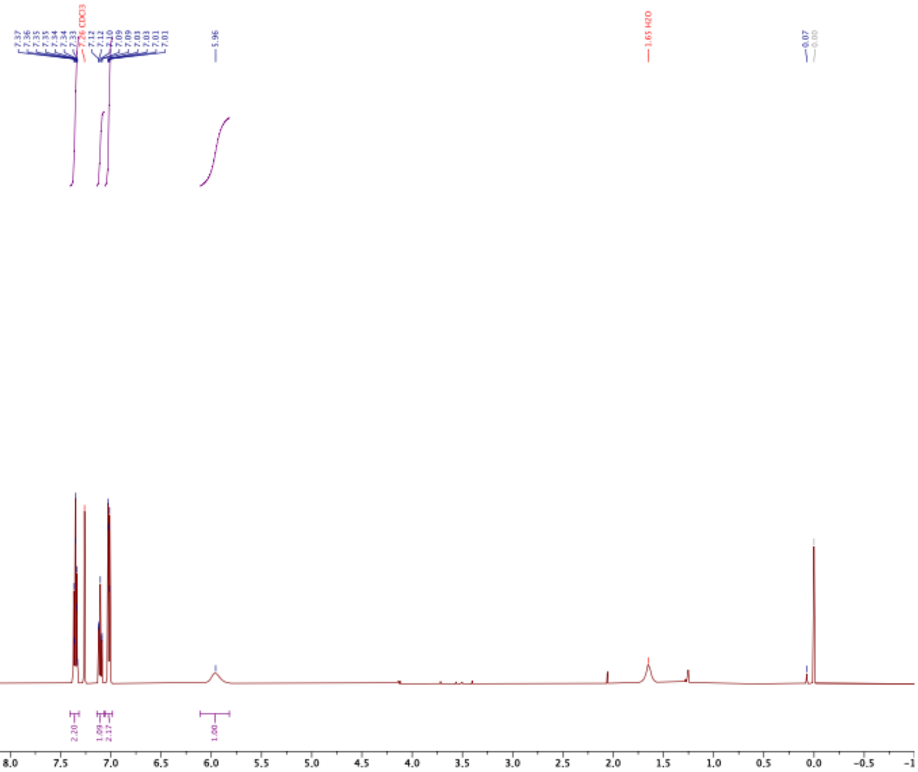

***N*-(4-Methoxyphenyl)cyanamide (8b)** [42]

^{1}H NMR (500 MHz, $CDCl_3$)

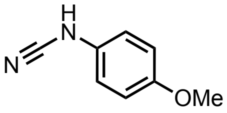

8b

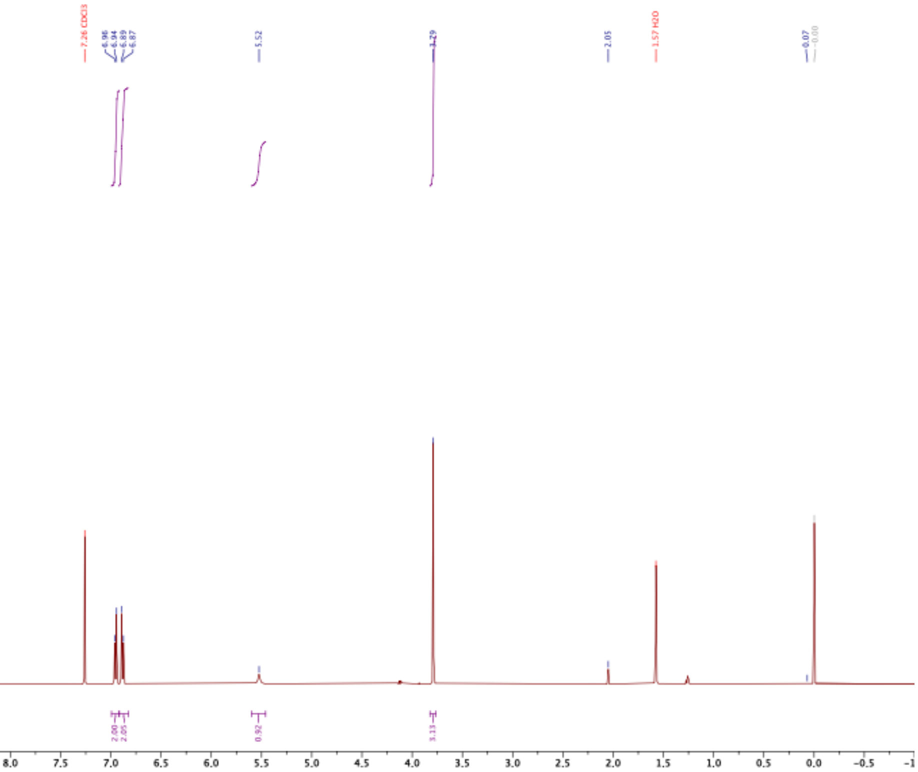

***N*-(4-Nitrophenyl)cyanamide (8c)**[43]

^{1}H NMR (500 MHz, DMSO)

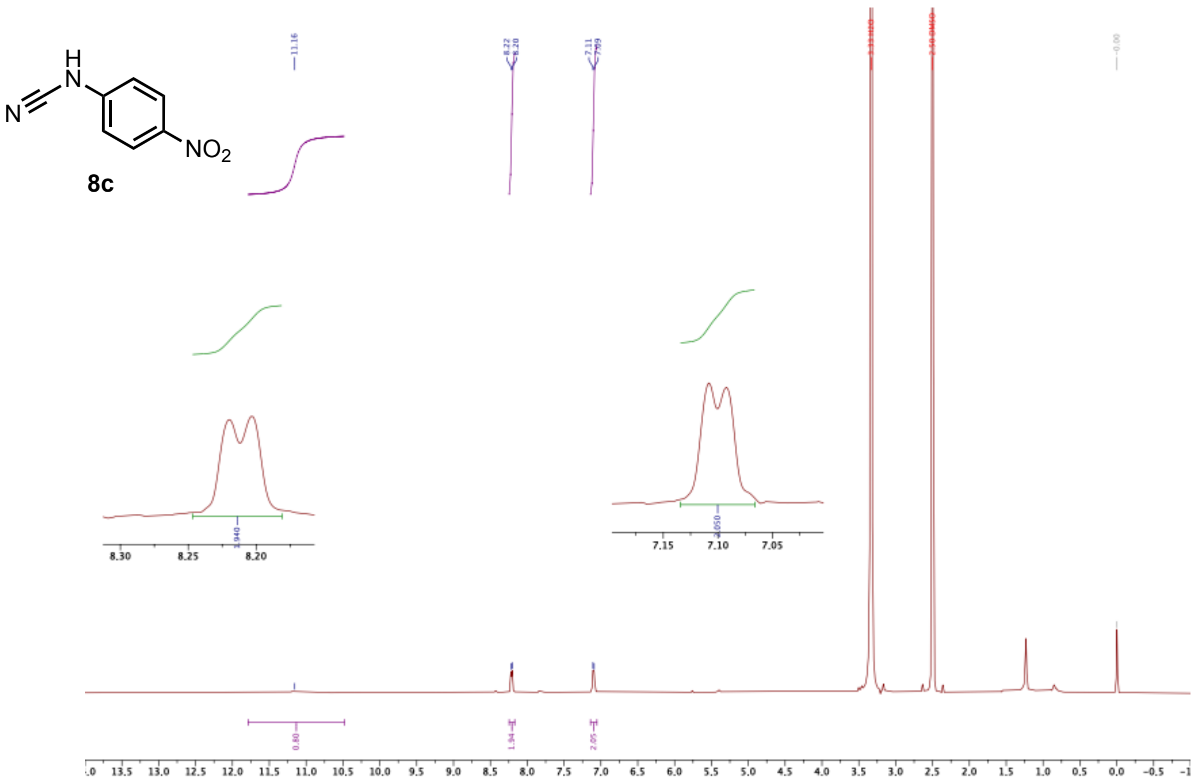

***N*-(2-Bromophenyl)cyanamide (8d)**[42]

^{1}H NMR (500 MHz, $CDCl_3$)

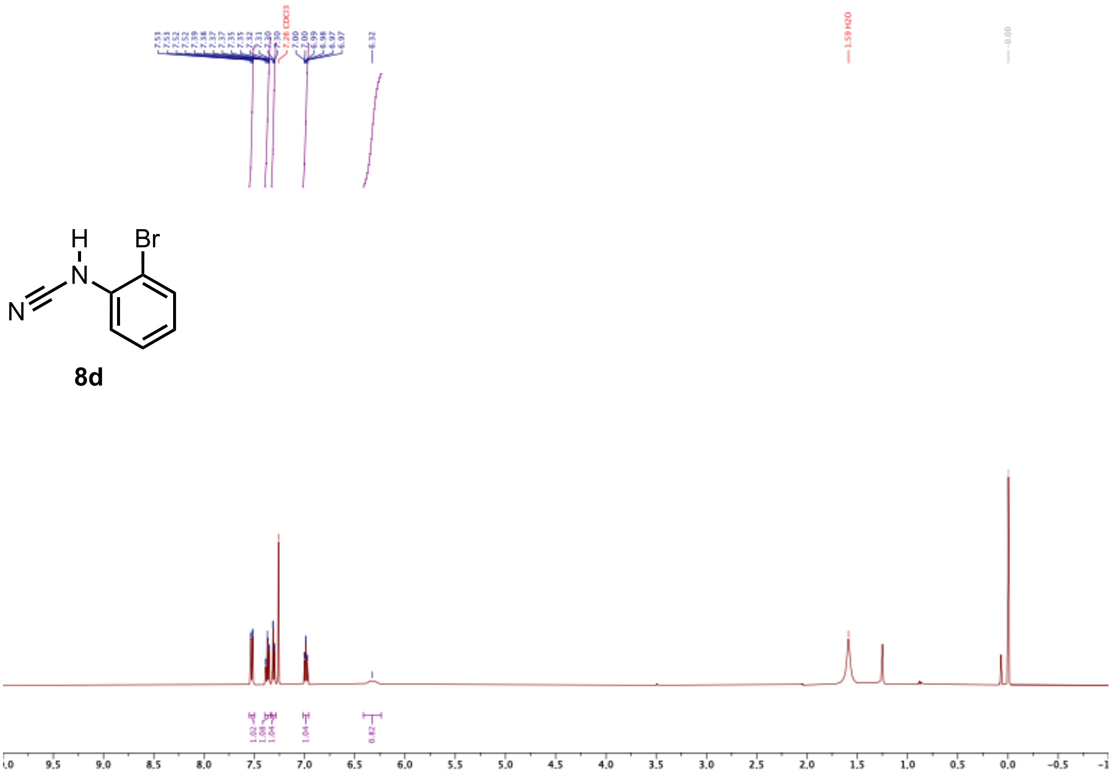

_N_-(3,5-Bis(trifluoromethyl)phenyl)cyanamide (8e)

^{1}H NMR (500 MHz, $CDCl_3$)

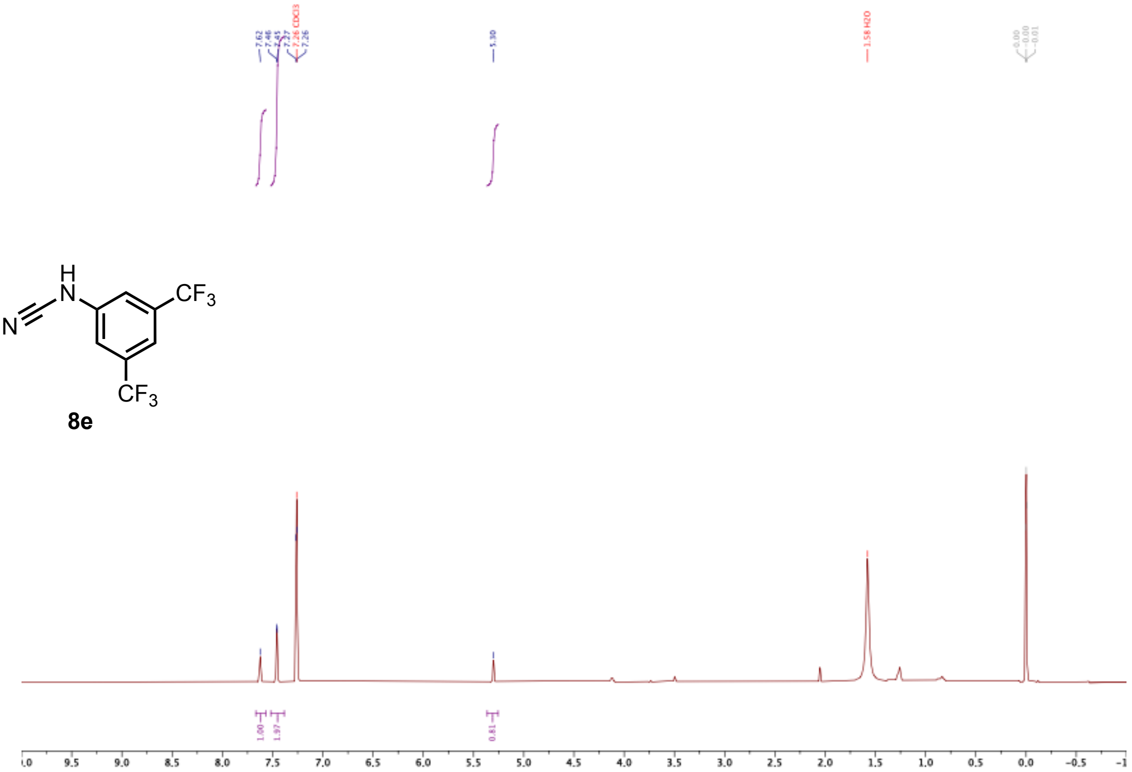

_N_-(3,5-Bis(trifluoromethyl)phenyl)cyanamide (8e)

^{13}C NMR (75 MHz, $CDCl_3$)

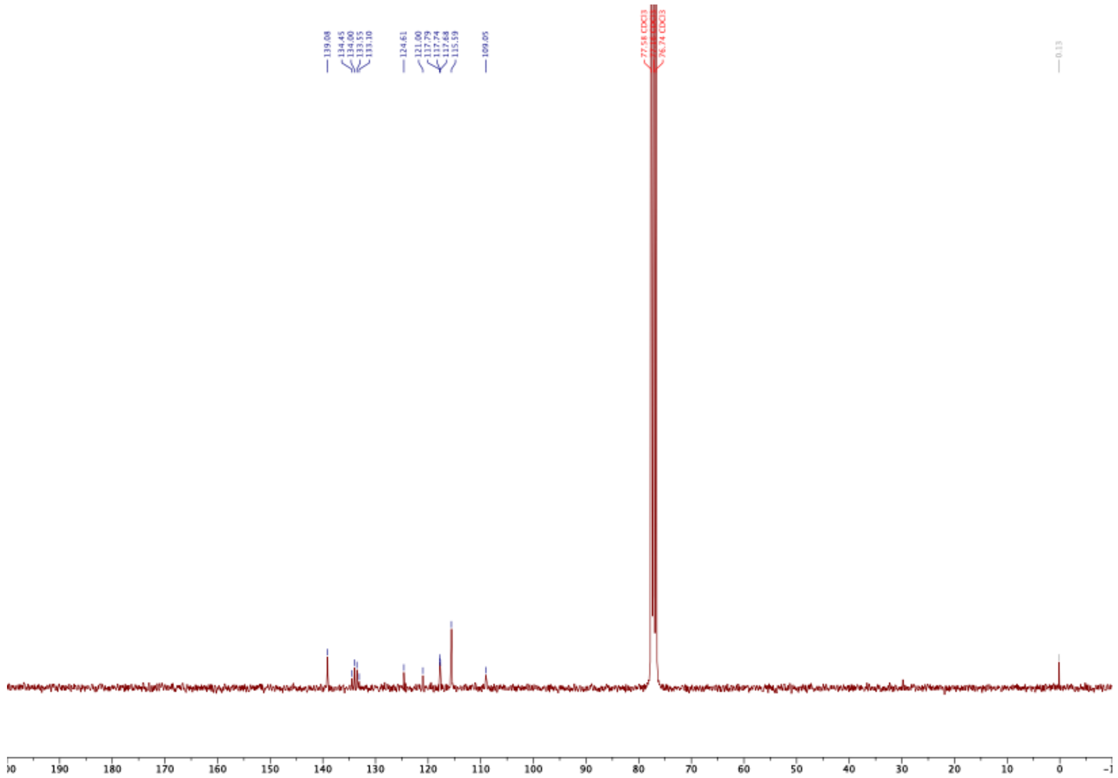

6-Methoxy-4-phenylisoquinolin-1(2*H*)-one (9) [34]

^{1}H NMR (500 MHz, $CDCl_3$)

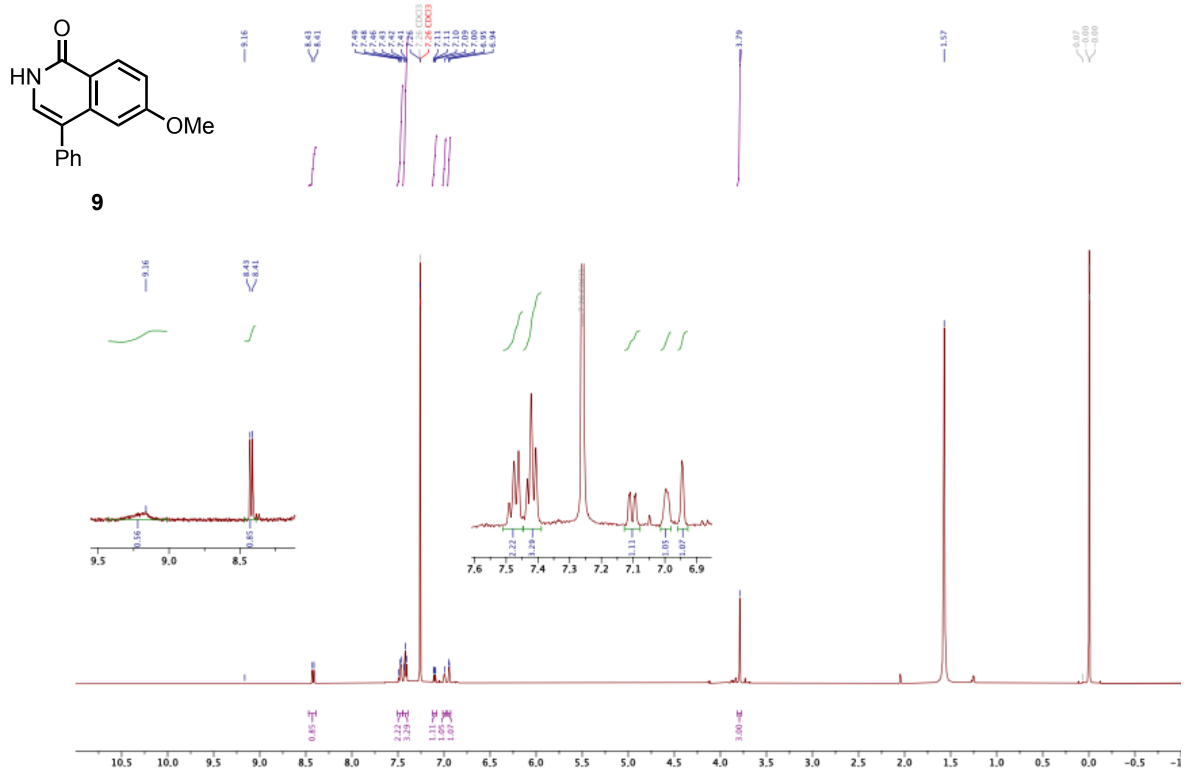

***N*-(Triphenyl-λ^5-phosphaneylidene)benzamide (10)**[44]

^{1}H NMR (500 MHz, $CDCl_3$)

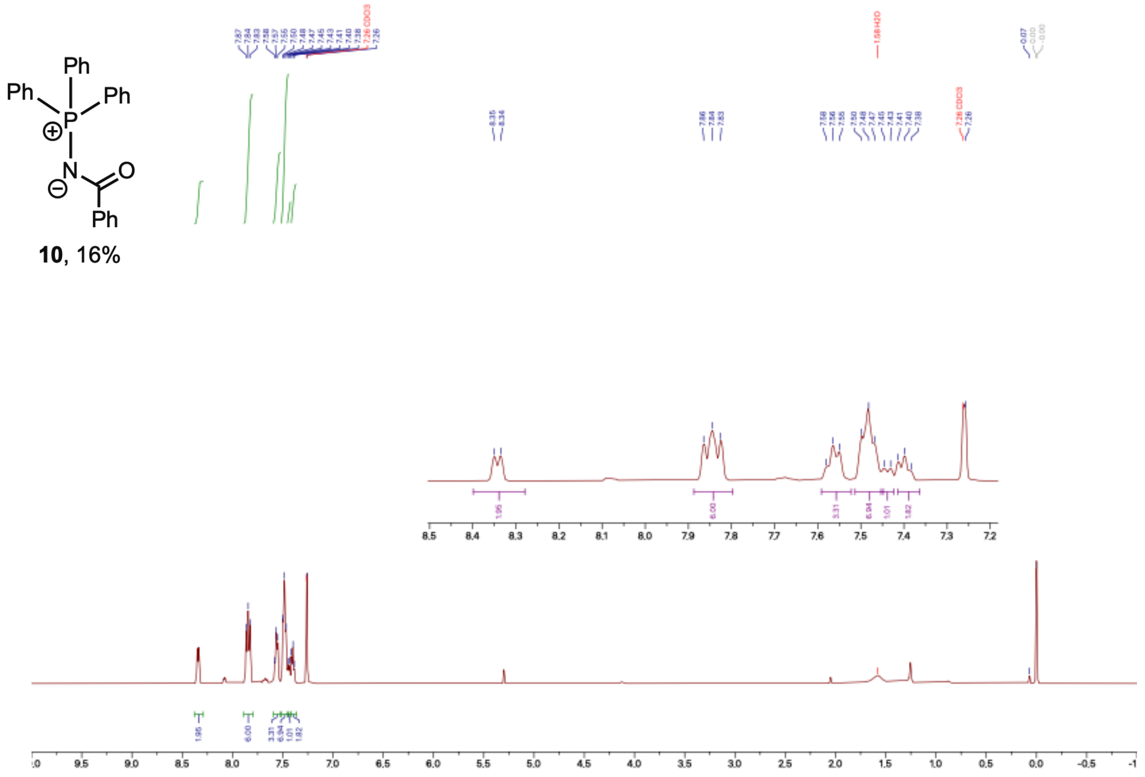

Supplement: Supplementary file 1 — Supporting Information [file CHEM-31-e202501045-s001.pdf]
